# Historia del Proyecto de Investigación en Servicios de Salud: Entrevista a Kerr White

**DOI:** 10.18294/sc.2023.47022

**Published:** 2023-11-22

**Authors:** Edward D. Berkowitz

**Affiliations:** 1 PhD en Historia. Profesor de Historia, George Washington University, Washington DC, EEUU. George Washington University George Washington University Washington DC USA

**Keywords:** Investigación sobre Servicios de Salud, Epidemiología de los Servicios de Salud, Atención Primaria de Salud, Ecología, Health Services Research, Epidemiology of Health Services, Primary Health Care, Ecology

## Abstract

Se presenta la entrevista realizada en 1998 a Kerr L. White (1917-2014), traducida al español. White fue un economista, epidemiólogo, estadístico, médico general, sanitarista, muy crítico de la separación entre la investigación, la clínica, la epidemiología, la salud pública y las políticas de salud. Fue uno de los fundadores de la investigación sobre los servicios de atención médica. Su preocupación básica fue la responsabilidad de la sociedad para asignar recursos de atención médica de la manera más apropiada para mejorar la salud de las poblaciones. La producción de White señala críticamente la ausencia de una mirada poblacional en la epidemiología aplicada a servicios y sistemas de salud, y en la evaluación del impacto social y económico de las prácticas, señalando además la deshumanización de los procesos de atención y cuidado. Sus trabajos han sido poco citados en los textos del campo de la salud, por lo que recuperamos su figura con el objetivo de difundir la vigencia de su pensamiento y su obra.

## Abstract (en)

The interview conducted in 1998 with Kerr L. White (1917-2014) is presented, translated into Spanish. White, an economist, epidemiologist, statistician, general physician, and health advocate, was highly critical of the separation between research, clinical practice, epidemiology, public health, and health policies. He was one of the pioneers in healthcare services research. His primary concern was society’s responsibility to allocate healthcare resources in the most appropriate way to improve the health of populations. White’s work critically addresses the absence of a population perspective in epidemiology applied to healthcare services and systems, and the lack of evaluation of the social and economic impact of practices. He also highlights the dehumanization of care processes. Despite being undercited in health literature, we reintroduce his figure with the aim of disseminating the relevance of his thoughts and contributions.

Berkowitz: Me gustaría indagar un poco acerca de su trayectoria personal y su enfoque en la investigación en servicios de salud. Comencemos hablando de usted. Me resulta interesante que, siendo canadiense, la mayor parte de su carrera profesional se ha desarrollado en EEUU. ¿Ha representado esto alguna problemática o consideración particular para usted?

White: No, no ha sido un problema para mí. Durante mi infancia y juventud visité EEUU con frecuencia. Mi madre era inglesa, pero tenía numerosos parientes estadounidenses. Mi padre era periodista y trabajaba para *The Times of London* y *The Economist*, así como para varios periódicos estadounidenses; por lo tanto, crecí con una perspectiva internacional. Recuerdo que en mi anuario de pregrado en la McGill University expresé que “soy un ciudadano del mundo, un internacionalista”. Trasladarme a EEUU no me generó preocupación alguna; aunque sí fue un poco más inquietante para mi esposa. Al concluir mi residencia y una beca posdoctoral en Medicina en McGill, tuve la opción de permanecer como miembro de la facultad. Sin embargo, opté por una excelente oportunidad en la University of North Carolina en Chapel Hill, donde pude combinar investigación, atención al paciente y docencia, áreas que me interesan profundamente.

Berkowitz: McGill siempre ha sido un lugar muy anglosajón. Supongo que la tensión entre los francoparlantes y los angloparlantes no era tan pronunciada en Canadá en aquel entonces.

White: No, la tensión ha aumentado con el tiempo. Históricamente, los francoparlantes estaban marginados y fuertemente dominados por la iglesia católica. En las décadas de 1930 y 1940, la educación clásica de orientación teológica que se proporcionaba a la mayoría de los canadienses franceses no los preparaba adecuadamente para el mundo comercial, intelectual y tecnológicamente avanzado que caracterizaría al emergente entorno global. Sin embargo, en décadas recientes, los canadienses franceses han demostrado una gran capacidad y habilidad para administrar su parte del mundo de manera excepcional. Espero que Quebec no se separe del resto de Canadá, ya que ambas partes perderían mucho.

Berkowitz: ¿Ha adquirido ya la ciudadanía estadounidense?

White: Soy un ciudadano estadounidense naturalizado y también un “súbdito británico” [se refiere a uno de los tipos de nacionalidad británica vigentes; hasta 1943, todos aquellos que tenían alguna relación cercana con Gran Bretaña eran llamados súbitos británicos (*British subject*)]. A ninguno de los dos gobiernos le agrada mucho la idea, pero legalmente es posible tener ambos pasaportes. Esto resulta muy útil si se desea realizar un viaje que incluya, por ejemplo, Japón, Hong Kong y Australia.

Berkowitz: Un aspecto inusual de su trayectoria académica es que obtuvo un posgrado en economía en Yale en 1941. ¿Cuál era su intención en ese momento? ¿Aún no había decidido ingresar al campo de la medicina?

White: Me especialicé en Economía y Ciencias Políticas en McGill y tenía un interés particular en el ámbito del transporte. Recibí una beca para el departamento de posgrado de economía de Yale y me centré en la economía y gestión del transporte. Un profesor que ejerció una gran influencia en mí fue Elliott Dunlop Smith. Él había trabajado en el departamento de personal de la Avery Dennison Company, donde observó la importancia del entorno laboral en la moral y la productividad de los trabajadores. Esto fue en 1940. Fue entonces cuando aprendí sobre el *efecto Hawthorne* que más tarde me influenciaría, y también sobre el impacto de la ocupación y el entorno laboral, incluidas las actitudes y los comportamientos de las áreas de gerencia en la salud de los empleados. Esto despertó mi interés en la medicina, hacia la cual me fui inclinando gradualmente. Mi primer empleo fue en la RCA Victor Company en Montreal como asistente junior de personal. Una de mis tareas cada viernes por la tarde era pagar a los trabajadores a destajo que ensamblaban radios para el ejército y el Departamento de Defensa. Estas personas, en su mayoría mujeres jóvenes, recibían un salario por hora. Les entregaba sobres con dinero que contenían $5,89, $17.21, etc., por una semana de trabajo. Se desmoronaban en lágrimas porque no tenían un ingreso estable y sus salarios eran miserables. Indignado por esta gran injusticia, tomé la iniciativa de ayudar a los trabajadores de RCA a organizar exitosamente un sindicato. Lideramos una huelga y pasé mucho tiempo con los dirigentes sindicales en hogares empobrecidos. Ganamos la huelga, negociamos un contrato, aumentamos sus salarios, establecimos un salario mínimo y otros diversos beneficios. Por supuesto, dejé RCA para hacer todo esto y, como resultado, fui investigado por la Real Policía Montada de Canadá (el equivalente canadiense de la CIA), aunque nunca derivó nada de ello. En el departamento de personal, trabajé con el departamento de salud de la empresa y observé cómo excluían a personas con discapacidades de salud; si alguien presentaba problemas de espalda o cualquier dificultad, era probable que lo despidieran. Esto me pareció injusto.

Berkowitz: ¿Se trata en parte de un problema relacionado con la compensación a los trabajadores por accidentes laborales, correcto?

White: Sí, pero en aquellos días no teníamos mucho de eso. Las experiencias en Yale y RCA son lo que despertaron mi interés por la medicina.

Berkowitz: En 1941, ¿sabía que tendría que unirse al ejército?

White: No, pero sospechaba que podría ser una posibilidad. Canadá participó en la Segunda Guerra Mundial desde su inicio, en 1939. Solicité ingreso a la escuela de medicina en 1942 y, aunque probablemente podría haber conseguido aplazar mi ingreso si me hubieran reclutado, decidí alistarme voluntariamente. Estuve en el ejército durante tres años.

Berkowitz: ¿Fue enviado al extranjero?

White: Estuve en Canadá y luego en Inglaterra. Estuve allí el Día D y durante todo el bombardeo con cohetes y bombas zumbadoras (*buzz bombing*). Ingresé a la escuela de medicina después de ser dado de baja del ejército en 1944.

Berkowitz: ¿Asistió a la escuela de medicina en McGill?

White: Así es.

Berkowitz: En la escuela de medicina, cuando llegó al punto donde tenía que decidir su especialidad, ¿qué pensó que sería?

White: Tenía intereses eclécticos en muchos aspectos de la medicina y escribí varios artículos en ese momento, incluido uno sobre educación médica integral y atención sanitaria en el que examiné una variedad de experimentos contemporáneos en la prestación de atención y en la educación médica. Estos fueron publicados en la revista médica estudiantil de McGill. Otro artículo trataba sobre enfermedades iatrogénicas; no estaba seguro de que los médicos siempre hicieran más bien que mal. Concluí en ese momento que el poder de la medicina estaba cambiando de la cirugía a la medicina interna y que, si quería tener alguna influencia, sería mejor capacitarse en medicina interna. Así que decidí eso, aunque también me atraía la práctica general. Nuestras hijas nacieron con el último médico general que tenía atribuciones obstétricas en el Royal Victoria Hospital, uno de los dos principales hospitales docentes de McGill. Pero ya estaba interesado en aspectos más amplios de la salud. Mi padre conocía a Jimmy McIntosh, un escocés como él, que en ese momento era el decano de la London School of Hygiene and Tropical Medicine. McIntosh le habló a mi padre sobre John Ryle en Oxford, la universidad de mi padre. Ryle había sido *regius profesor* de Medicina en Cambridge, que como saben es uno de los puestos más importantes en la medicina académica en Gran Bretaña, dado que su designación es aprobada por la Corona británica. Se convenció de que las escuelas de medicina deberían adoptar una perspectiva más amplia basada en la población, además del énfasis puramente clínico que prevalecía. El *trust Nuffield* [organización caritativa privada que financia proyectos en salud] estableció una cátedra y un Instituto de Medicina Social; el primero de su tipo en cualquier lugar, en la Universidad de Oxford, y Ryle cambió de universidades y disciplinas para convertirse en el primer profesor de Medicina Social en Oxford. Inició estudios sobre la distribución de los servicios de salud y de las enfermedades en la población. Su trabajo me intrigó y estaba en proceso de negociar para pasar un año de beca o algo así con él, pero lamentablemente falleció y eso nunca se concretó. Ryle escribió un pequeño volumen notable llamado *Changing disciplines*, basado en una serie de conferencias que dio durante una gira patrocinada en EEUU y Canadá por la Fundación Rockefeller, para la cual, por coincidencia, trabajé recientemente. También escribió varios volúmenes sobre medicina clínica que me parecieron notablemente perspicaces. Discutió la historia natural de los síntomas comunes, conceptos que fueron la base para determinar qué es “normal” y una variedad de otros temas que cuestionaban la sabiduría clínica recibida e invitaban a una exploración más profunda. Por primera vez me encontré con un médico académico distinguido, que no solo actuaba como un profesor autoritario, sino como parte de una nueva generación que enfatizaba la importancia de buscar una base científica para las intervenciones clínicas. Cuando llegó el momento de la pasantía, podría haberme quedado en McGill y me dijeron que más tarde debería ir a Boston o Baltimore para recibir capacitación adicional. Me complació el interés de la facultad en mí, pero dije que no quería pasar un año corriendo con una jeringa sacando sangre para que otros hicieran estudios en el laboratorio. Quería aprender medicina clínica de primera categoría. En retrospectiva, la atención clínica en McGill fue muy superior a cualquier cosa que haya encontrado en cualquier hospital docente estadounidense, incluido Hopkins. Quería ir a un lugar donde realmente practicaran medicina clínica a tiempo completo. Las tres hermanas, como se les conocía, tenían esa reputación. Son el Mary Imogene Basset Hospital en Cooperstown, New York, el Mary Fletcher Hospital en Burlington, Vermont, y el Mary Hitchcock Hospital en Hanover. Un amigo mío, que ya ha fallecido, y yo decidimos juntos que optaríamos por el Mary Hitchcock Hospital. Ambos fuimos aceptados allí y realmente obtuvimos una formación clínica excelente. Nunca he visto atención clínica de tan alta calidad en ningún otro lugar. Las cosas pueden haber cambiado, ya que esto fue hace mucho tiempo, pero recibimos mucha tutoría individualizada.

Berkowitz: ¿Hanover no es una zona bastante rural?

White: Lo es.

Berkowitz: ¿El hospital está afiliado a Dartmouth?

White: Sí. Ahora Dartmouth-Hitchcock es un centro médico bastante sustancial, aunque en aquellos días era más modesto. Tiene una amplia área de captación en New Hampshire y Vermont y los estados adyacentes. Pero tienes razón, es básicamente rural en comparación con los centros urbanos de New York o Boston. Mi antiguo alumno, Jack Wennberg, tiene un gran centro de investigación en Dartmouth. Realiza todos sus estudios poblacionales de renombre mundial sobre las enormes variaciones geográficas en diagnósticos e intervenciones clínicas desde ese punto de vista. La ubicación en un entorno semirural no tiene nada que ver con la atención, educación o investigación de primera categoría. Después de mi residencia en medicina interna en Dartmouth, regresé a McGill con una beca en medicina. También he tenido algo de formación en psiquiatría. No fue una formación completa en psiquiatría, pero asistí a seminarios y participé. El profesor John S. L. Browne, el presidente de Medicina en McGill, me invitó a quedarme, pero tenía que mantener a mi esposa y dos hijos y habría tenido que pasar la mayor parte de mi tiempo yendo a diferentes hospitales para ejercer la práctica privada, lo cual estaba bien, pero me parecía que implicaba un gasto excesivo de energía para un ingreso bastante modesto. JSL (como se lo conocía a Browne) fue de gran ayuda al presentarme a Lester Evans del Commonwealth Fund en New York. Él generosamente se interesó en mí. De hecho, mi visita al Commonwealth Fund en 1949 inició una relación que se ha extendido durante casi cincuenta años. Evans discutió dos o tres posibles aperturas que pensó que podrían ofrecer oportunidades. Una era la University of North Carolina en Chapel Hill, donde fui nombrado profesor asistente de medicina interna y comencé mi carrera académica formal.

Berkowitz: Para entonces, ya estabas en la vía académica. ¿Fue algo que decidiste en algún momento? Por un lado, estás haciendo este trabajo clínico en Dartmouth, pero decidiste que vas a ser un académico.

White: Bueno, sí. De hecho, hice algunas investigaciones mientras estaba en Hitchcock y publiqué dos o tres artículos desde allí. No estoy seguro de haber elegido la vía académica hasta que JSL me alentó. No lo había pensado conscientemente antes de eso, aunque indirectamente, por supuesto, me habían alentado a hacerlo en McGill cuando me gradué. En Dartmouth, me pidieron que me quedara en la Clínica Hitchcock y en la facultad, pero habría sido principalmente un trabajo clínico en medicina, aunque había enseñanza involucrada y posiblemente alguna investigación. De todos modos, pensé que debería recibir más formación y regresé a McGill para el año de beca. No puedo decir que tomé una decisión consciente en la escuela de Medicina o incluso antes de eso para embarcarme en una carrera académica.

Berkowitz: Cuando fuiste a Carolina del Norte, ¿no querías estar en salud pública? ¿Se te pasó por la cabeza?

White: No. En McGill teníamos a un ex funcionario de salud de Ontario que era el profesor de Medicina Preventiva y Salud Pública. Dio una serie de conferencias desalentadoras sobre cómo cavar un pozo para letrinas y cómo enlatar tomates para que no contraigas botulismo y otros temas sombríos. Pensé que debía haber algo más en este aspecto de la medicina que lo que este tipo podía exponer. De hecho, fui a verlo un par de veces y le dije: “¿*Qué pasa con este tema de la salud pública*?” y él dijo: “*Bueno, es tal y tal*”. Era un personaje muy poco inspirador. Tomé un juramento en ese momento de que no tendría mucho que ver con la salud pública tal como la practican este tipo de personas. Así que la respuesta corta es no, no consideré la idea de una Maestría en Salud Pública. Consideré la idea de pasar el año con Ryle en su nuevo Instituto de Medicina Social en Oxford si hubiera sido factible pero, como mencioné anteriormente, murió inesperadamente.

Berkowitz: ¿Carolina del Norte no se había convertido en una escuela de medicina de cuatro años?

White: Sí, había sido una escuela de medicina de dos años.

Berkowitz: Es interesante que un centro académico importante hasta la década de 1940 fuera solo una escuela de dos años.

White: Era una escuela de dos años y se convirtió en una escuela de cuatro años en 1952-1953; de hecho, fue la primera escuela nueva de cuatro años después de la Segunda Guerra Mundial. Antes, cuando era una escuela de dos años, no había profesorado clínico, así que fuimos algunos de los primeros profesores clínicos allí y floreció. Tampoco había una Escuela de Salud Pública allí. Esta última se inició alrededor de 1954 por Ed McGavran en el sótano del edificio de la Escuela de Medicina. Me hice amigo de muchos de sus nuevos profesores, en particular de John Cassel, un epidemiólogo social que estaba muy adelantado a su tiempo; un tipo notable. Provenía de un grupo de refugiados del *apartheid* en Sudáfrica. Eran discípulos de Sidney Kark, un pionero en el desarrollo del concepto de atención médica orientada a la comunidad. Escribió varios libros sobre el tema. Ahora está en Israel. Creo que regresó a Sudáfrica durante un par de años y ha sido profesor visitante en varias universidades. Lo vi por última vez hace diez o doce años. De hecho, él y su esposa fueron mis pacientes cuando todos estábamos en Chapel Hill. Había venido allí como el primer jefe de su departamento de epidemiología. El grupo de Kark, todos los cuales emigraron de Sudáfrica, incluía a varias personas notables. Abe Adelstein fue jefe de estadísticas de salud en el Reino Unido; Harry Phillips se convirtió en una autoridad líder en rehabilitación en Boston; Mervin Susser de Columbia es un epidemiólogo destacado y, por supuesto, John Cassel. John y yo nos hicimos grandes amigos; murió prematuramente, fue muy triste. Bernie Greenberg, otro amigo y colega, fue jefe de bioestadística en la Escuela de Salud Pública. Bernie luego se convirtió en decano de la Escuela de Salud Pública. Aprendí mucho de él. Todos éramos buenos amigos y organicé para que John y Bernie enseñaran en la Escuela de Medicina y solía participar en sus seminarios en la Escuela de Salud Pública y todos colaboramos en la investigación. Pero me había comprometido a mí mismo a no unirme a la facultad de una Escuela de Salud Pública. Sentía que el verdadero futuro era hacer que los médicos pensaran de manera más amplia sobre la enfermedad y la atención médica. La mayor influencia en mí, en ese momento, fue que la escuela recién ampliada, cuando se abrió, había patrocinado una conferencia en la que Jerry Morris de Londres era la atracción estelar. Era jefe de la Unidad de Investigación de Medicina Social del Consejo de Investigación Médica del Reino Unido en el Hospital de Londres. Desafortunadamente, el seminario tuvo lugar antes de mi llegada a Chapel Hill, pero escuché todo al respecto. Cecil Sheps estaba allí. Aunque más tarde se mudó a Boston y luego a Pittsburgh, regresó a la University of North Carolina y finalmente se convirtió en vicecanciller. Un artículo de Morris publicado en el *British Medical Journal*, titulado “*The uses of epidemiology*”, me impresionó mucho. Morris demostró que los conceptos y métodos epidemiológicos podrían aplicarse para comprender y evaluar mejor la prestación de servicios de salud. Entre otras cosas, enfatizó la importancia de determinar si las intervenciones médicas de todo tipo hacían más bien que mal. Examinó el impacto en la salud y la enfermedad de una serie de factores nutricionales, ambientales, ocupacionales y sociales. Mientras todo esto estaba sucediendo, la Fundación Rockefeller, representada por John Grant, uno de sus oficiales más creativos, había ofrecido construirnos un edificio y financiar una práctica grupal de profesores prepagada para demostrar su viabilidad para brindar atención médica, realizar investigaciones y educar a los estudiantes de medicina. Todo esto fue en 1953-1954. Tengo una copia de la propuesta original presentada por John Grant a la Escuela de Medicina de la Universidad de Carolina del Norte. Pero este plan subversivo fue rechazado por la Facultad de Medicina. Realmente estaba fuera de lugar en aquellos días.

Berkowitz: Solo había unos pocos de ellos, como el Group Health en Washington, Kaiser en California.

White: Correcto. Pero Grant pensó que la práctica grupal y el prepago se convertirían en el modo preferido de práctica en el futuro y que un centro académico debería asumir esto y desarrollarlo, pero las sociedades médicas locales y estatales estaban en armas y amenazaron con que ocurrirían cosas terribles si estas ideas eran adoptadas por la nueva escuela de medicina. Hicieron presión en la legislatura estatal para oponerse al plan con el resultado de que la escuela de medicina se echó atrás; temían que les recortaran los fondos estatales. Ese ejercicio también tuvo una poderosa influencia en mí.

Berkowitz: Tengo curiosidad por esto. Antes de que existiera esta escuela de cuatro años en Chapel Hill, ¿hubo alguna escuela de medicina de cuatro años en Carolina del Norte?

White: Duke probablemente tenía una escuela de medicina de cuatro años y probablemente Bowman-Gray, pero no estoy seguro de eso. Duke casi seguro que estaba funcionando entonces, sí.

Berkowitz: Entonces, la idea era que la educación médica se la consideraba como algo de la enseñanza privada. En la Missouri University, sé que tenían una escuela de dos años durante mucho tiempo. Tal vez esa era la idea, que no se esperaba que la escuela estatal tuviera una escuela de medicina.

White: No estoy seguro de esa historia. No sé cuántas universidades estatales tenían escuelas de medicina. California debe haber tenido algunas, pero simplemente no lo sé.

Berkowitz: Eso es interesante. Cuando pienso en Carolina del Norte, una de sus grandes cosas era la sociología rural. ¿La medicina también estaba orientada hacia lo rural?

White: En efecto, hasta cierto punto. Realizamos numerosas investigaciones con Reuben Hill, miembro del Departamento de Sociología de la University of North Carolina, y con Harvey Smith, un sociólogo de la Escuela de Medicina de la University of North Carolina. También estaba Harry Martin, otro sociólogo, a quien solía llevar conmigo en las recorridas clínicas. Una vez se desmayó cuando vio un paciente con un poco de sangre encima. Trabajamos estrechamente con todos ellos. Solía asistir a las reuniones semanales del Instituto de Sociología de la University of North Carolina; creo que ese era el nombre de la entidad. Nuestro grupo se consolidó principalmente alrededor de Bernie Greenberg y yo. Luego estaba Dan Martin, un internista, que trabajó conmigo como becario de investigación; posteriormente, se convirtió en una figura destacada en la Clínica Trover en Kentucky. También contábamos con Frank Williams, otro miembro del Departamento de Medicina de la University of North Carolina, quien más tarde se convirtió en director del Instituto Nacional sobre el Envejecimiento. Era un poco más joven que yo. También estaba Bob Huntley, quien se convirtió en presidente y profesor de Medicina Comunitaria en Georgetown. Teníamos un núcleo de curiosos colegas docentes de diversas disciplinas y comenzamos a hacer lo que llamamos “investigación en atención médica”. Alrededor de 1955, la Ley Hill-Burton incluyó un millón de dólares para investigaciones sobre lo que llamó “instalaciones hospitalarias y médicas”. Vi un aviso de esto en el *Journal of the American Medical Association* (JAMA) y pensé que era el tipo de financiamiento que necesitábamos conseguir, así que presentamos una solicitud de subvención -el número era W74, lo recuerdo bien-, y nos otorgaron un paquete considerable de fondos. Esto nos inició en un estudio sobre las derivaciones de pacientes de médicos generales (ahora llamados médicos de familia) a consultores o especialistas en centros médicos universitarios en Carolina del Norte. Había dos preguntas que me intrigaban: ¿estamos haciendo más bien que mal en lo que hacemos por los pacientes? Y, en segundo lugar: ¿estamos respondiendo a las necesidades reales, expectativas y deseos del paciente en el encuentro clínico? Estas me parecían preguntas para nada descabelladas, pero aparentemente no se les había ocurrido a muchos de los académicos médicos de esa época. Así que nuestro grupo de investigación se embarcó en el estudio de las derivaciones. Bernie Greenberg, el estadístico, nos ayudó inmensamente. Realizamos una muestra probabilística de todos los médicos generales del estado y entrevistamos a casi todos ellos. Luego tuvimos pacientes índice que fueron derivados a nuestra clínica general en la University of North Carolina por cada uno de los médicos generales en nuestro estudio. Los médicos fueron entrevistados por nosotros y los pacientes fueron entrevistados tanto en la clínica general de la universidad como en su hogar por un trabajador social que formaba parte de nuestro equipo. Seguimos a cada uno y registramos, entre otros factores, los diferentes patrones de derivación a Duke, la University of North Carolina y otros centros médicos. Tuvimos una tasa de respuesta muy alta, de aproximadamente el 96%, según lo recuerdo. Este fue el primer estudio real de perfiles de derivación que se había realizado. Publicamos cuatro o cinco documentos sustanciales de un tipo u otro durante un período de varios años. El más conocido de estos es “*The ecology of medical care*”, publicado en el *New England Journal of Medicine* en 1961. En ese artículo, entre otros asuntos, introdujimos el término “atención médica primaria”. Nuestro estudio de derivación ha sido replicado numerosas veces con resultados básicamente idénticos. El estudio más extenso se completó hace unos años en Canadá. Encontraron esencialmente las mismas cosas que nosotros. Documentamos una falta de comunicación masiva entre el médico que derivaba, el paciente, el estudiante de medicina en nuestra clínica, el médico tratante y, a veces, de otros participantes en el proceso de derivación. El médico general derivó al paciente por una razón, el o la paciente pensó que estaba siendo derivado por alguna otra razón, el estudiante de medicina determinó que el problema era una afección completamente diferente, y luego el residente y el médico tratante consideraron que el problema más importante era otra cosa. Este último podría estar interesado en enfermedades de la tiroides y logró encontrar un nódulo tiroideo, mientras que el paciente estaba realmente deprimido, aunque había sido derivado por el médico general debido a “dolor de espalda”. Simplemente una falta de comunicación masiva. Esto parecía tener varios frentes.

Berkowitz: ¿Es realmente falta de comunicación? Eso suena más a propaganda para la medicina de rehabilitación. Siempre hablan de la coordinación de la atención y ese tipo de cosas.

White: No, no. Ese fue solo un ejemplo, pero había muchos otros tipos de mala comunicación.

Berkowitz: Pero el punto es que el paciente tenía todas esas cosas mal, ¿verdad? Simplemente dependía de qué médico estuviera mirando al paciente.

White: Sí, dependiendo de qué médico estuviera mirándolo. Sí, supongo que todas esas cosas podrían estar mal de una forma u otra, pero responder a las expectativas del paciente debería estar en la parte superior de la lista.

Berkowitz: El médico de rehabilitación diría que lo que necesita es un coordinador del equipo. No sé cuánto de eso está de moda ahora, pero fue una gran idea a finales de la década de 1940, y principios de la década de 1950.

White: En nuestros artículos enfatizamos la necesidad de que todos tengan un médico general para coordinar su atención. Fue entonces cuando introdujimos el término “atención médica primaria” que mencioné anteriormente. He hecho una carrera explicando el papel esencial de la “atención primaria” en cualquier sistema de atención médica bien equilibrado. La atención primaria es de importancia central cuando se consideran las necesidades de toda la población. Debería tener una alta prioridad al diseñar un plan de estudios médicos, seleccionar proyectos de investigación y organizar servicios de atención médica. No sé si te has encontrado con nuestro artículo “*The ecology of medical care*” en el que se exponen estas ideas.

Berkowitz: Sí, fue publicado en 1961 en el *New England Journal of Medicine*. ¿Es correcto?

White: Ilustramos nuestros hallazgos con una serie de cinco cajas anidadas. Atribuimos la idea de las cajas anidadas a un artículo de John y Elizabeth Horder que encontré cuando estuve en Inglaterra. Habían examinado su propia práctica y descrito la distribución de pacientes que iban al hospital, eran derivados a consultorios, etc. Pensé que podríamos hacer el mismo tipo de análisis para poblaciones enteras y así lo hicimos.

Berkowitz: Esta subvención que obtuviste de Hill-Burton, dijiste que era W74. ¿Era ese el nombre de tu propuesta o el número de la subvención?

White: Era el número de la subvención. Creo que el título era algo así como “Un estudio de los patrones de derivación de pacientes en Carolina del Norte”.

Berkowitz: Ahora que sabes un poco más sobre el proceso político, ¿por qué había ese dinero? ¿Fue idea de Fogarty? ¿Fue idea de Hill? Fogarty era muy partidario de asignar fondos en Hill-Burton.

White: Fueron Louis Block y su jefe, Jack Haldeman, del Servicio de Salud Pública de EEUU, quienes iniciaron la idea. Block, creo, había sido consultor de hospitales y habló con Fogarty y con Hill para que incluyeran los fondos en la asignación de Hill-Burton. Hasta donde sé, ese fue el dinero inicial para el campo que finalmente se conoció como “investigación en servicios de salud”. Había estado haciendo investigaciones cardiovasculares y estaba interesado en el efecto de las emociones en las enfermedades cardiovasculares, particularmente en la insuficiencia cardíaca. Habíamos realizado una serie de estudios de laboratorio, entrevistas exhaustivas con pacientes y encuestas basadas en la población. De hecho, nuestra primera solicitud de subvención fue rechazada por la sección de estudios cardiovasculares de los National Institutes of Health. El National Heart Council, a cargo de Tinsley Harrison, autor de uno de los principales libros de medicina, decidió que mis ideas no eran tan descabelladas. Vino a Chapel Hill liderando él mismo un grupo para visitar el sitio. Desde su experiencia clínica, pensaba que teníamos algo. Nuestros hallazgos de que la angustia emocional puede desempeñar un papel importante en la precipitación de la insuficiencia cardíaca han sido confirmados posteriormente por otros estudios. Obtuvimos la subvención para realizar trabajos de laboratorio sobre los efectos de las emociones en la función venosa y cardíaca. Presentamos un artículo en una reunión anual de la American Heart Association que atrajo una atención considerable. La historia fue publicada en numerosos periódicos de todo el país y en la revista *Time*. Una historia de televisión recibió una amplia difusión, pero nunca la vi. Mi interés en la insuficiencia cardíaca no se limitaba al laboratorio o al lecho del paciente, incluía la pregunta: “¿es este un problema grande o pequeño en la población en general?” Había muy pocos datos disponibles. No pude encontrar ninguna estadística al respecto porque en ese momento no estaba clasificado fácilmente en la Clasificación Internacional de Enfermedades (CIE). Uno de los estudiantes de la Escuela de Salud Pública, Michel Ibrahim, quien ahora es el decano de la Escuela de Salud Pública en Chapel Hill, hizo su tesis doctoral conmigo. Realizamos un estudio epidemiológico de base poblacional que se publicó en *Annals of Internal Medicine*, en el que mostramos la distribución de la insuficiencia cardíaca en toda la población por primera vez. Descubrimos que el uno por ciento de la población lo padece en un momento dado y que el 10% de los mayores de 65 años lo están experimentando. Cuando fui a Vermont, repetimos el estudio y encontramos los mismos tipos de distribuciones. Esos estudios, los primeros de su tipo, se incorporaron en el informe histórico de Michael DeBakey sobre enfermedades del corazón, accidentes cerebrovasculares y cáncer en la década de 1960. Al estudiar este problema en el laboratorio, la clínica o el lecho del paciente y la población, creo que mostramos que los tres enfoques pueden ser útiles para comprender la génesis, la historia natural y la distribución de los problemas de salud.

Berkowitz: Cuando dices de base poblacional, ¿te refieres a que observas datos de un gran número de personas?

White: No es solo una cuestión de grandes números. Los estudios de base poblacional abarcan una población base que generalmente es toda la población dentro de una jurisdicción geopolítica definida, pero puede ser un subconjunto de esa población. Los estudios pueden realizarse a partir de muestras aleatorias extraídas de la población índice para que el sesgo de selección se minimice. Aprendí de Jerry Morris, a quien mencioné anteriormente, cuando pasé un año con él en 1959-1960, sobre las tres formas en que se pueden examinar los problemas de salud. Puedes trabajar en el laboratorio, que está bien para ciertos problemas y preguntas; puedes estudiar a los pacientes en la clínica o el hospital en el encuentro uno a uno entre el médico y el paciente; y luego puedes hacer estudios epidemiológicos de la población. Uno no es bueno o malo, correcto o incorrecto, blando o duro. Depende de la pregunta y del problema que estés abordando. Algunas cosas se pueden observar mejor en una población. Puedes ver cuáles son las distribuciones, porque a menudo se necesitan grandes números para detectar los factores de interés, y también quieres incluir no solo a aquellos que buscan atención médica y acuden a un médico o un hospital, sino también a aquellos que no buscan atención, que pueden o no necesitar atención o tener alguna discapacidad.

Berkowitz: Entonces, si desea realizar un estudio epidemiológico, necesita hacer un estudio de base poblacional.

White: Correcto. Sin embargo, hay excepciones.

Berkowitz: ¿Un estudio de enfermedad? ¿Quizás realice un estudio clínico?

White: La noción que se debería utilizar un solo enfoque para comprender las enfermedades es uno de los problemas que ha resultado de la separación de las escuelas de salud pública de las facultades de medicina. Esta división fue obra de la Fundación Rockefeller en 1918, cuando los bienintencionados oficiales de ese día eliminaron la perspectiva poblacional de las perspectivas clínicas y de laboratorio o biomédicas (como se las llama hoy en día). Un libro que escribí hace varios años, *Healing the schism: epidemiology, medicine, and the public’s health*, aborda la historia de esta desafortunada separación. Los clínicos empleaban previamente los tres enfoques, pero de repente la perspectiva poblacional fue segregada en dominios separados en las escuelas de salud pública, comenzando por Johns Hopkins. Creo que esta iniciativa bien intencionada hizo más daño que beneficios. Resultó que investigadores y docentes familiarizados con la perspectiva poblacional se separaran intelectual y culturalmente de investigadores y docentes biomédicos que estaban realizando avances extraordinarios en nuestra comprensión de los procesos de la enfermedad. Estos últimos, sin embargo, contribuían mucho menos a nuestra comprensión de los múltiples factores sociales, conductuales, psicológicos, ambientales, ocupacionales y nutricionales que influyen en el mantenimiento de la salud y la génesis de la mala salud. Iba a hablarles sobre el artículo de ecología. Teníamos subvenciones tanto de la Fundación Rockefeller como del Commonwealth Fund para apoyar nuestra clínica general. Estábamos brindando atención médica a la población general en un esfuerzo por restablecer el papel del médico general. Chuck Burnett, presidente de nuestro Departamento de Medicina Interna y un fuerte partidario de nuestro trabajo, estaba enfermo y su segundo al mando, Lou Welt, estaba a cargo. Lou era un nefrólogo de renombre y escribió uno de los textos clásicos sobre el tema. Era muy reduccionista y, para algunos de nosotros, parecía volverse cada vez más estrecho en su pensamiento sobre la medicina y la educación médica. Recuerdo haber ido a verlo y decirle: “*los funcionarios del Commonwealth Fund vienen de New York para hacer una revisión de nuestra enseñanza e investigación para determinar si deben volver a financiarnos. Necesitamos escribir un informe*”. “*Oh*”, dijo Lou, *“¡simplemente diles que gastamos el dinero!*” Su respuesta me enfureció, y recuerdo haber vuelto a mi oficina y decir: “*Estos tipos altamente especializados simplemente no entienden cuál es el problema. No es solo la atención terciaria en la que hay que enfocarse en la facultad de medicina, sino que están todas esas personas allá afuera con otros tipos de discapacidades y problemas que no necesariamente acuden a los centros médicos universitarios, pero requieren comprensión y atención. De alguna manera, tenemos que preparar a los médicos para que también atiendan sus necesidades*”. Así que tomé datos de base poblacional de EEUU y Gran Bretaña y los datos de nuestros propios estudios de referencia, creé el diagrama de cuadrados anidados y escribí “*The ecology of medical care*”. Bernie Greenberg, mi amigo bioestadístico, ayudó a obtener el dimensionamiento de los cuadrados matemáticamente correctos. Ese es el artículo en el que introdujimos el término “atención médica primaria”.

Berkowitz: ¿Para significar qué, exactamente?

White: Para significar la atención médica fundamental, básica, inicial y continua que proporciona la base de cualquier sistema de atención médica equilibrado, efectivo y eficiente, organizado para satisfacer las necesidades de toda la población. Se necesita atención primaria y atención secundaria, y también se necesita atención terciaria. A finales de 1960 o principios de 1961, Richard Scott, un médico general que conocí por primera vez en Escocia en 1959, había establecido en la University of Edinburgh la primera Unidad de Enseñanza de Práctica General en el mundo y fue apoyado por la Fundación Rockefeller. La Fundación estableció una unidad similar en la University of Manchester bajo la dirección de Robert F.L Logan, a quien también conocía muy bien. John Brotherston, quien era entonces decano de la Facultad de Medicina en Edimburgo, había establecido la unidad de Scott, creando una cátedra y haciendo de Scott el primer profesor Sir James Mackenzie de Práctica General. Fue la primera cátedra de Práctica General en el mundo. James Mackenzie es el gurú y héroe de los médicos de familia y médicos generales en todo el mundo. Su historia es importante; cada estudiante de medicina debería leer sus dos biografías: *Beloved physician* de McNair Wilson y *Sir James Mackenzie MD: General Practitioner 1883-1925* de Alex Mair. En cualquier caso, Scott y yo estábamos preparando documentos de trabajo para un Comité de Expertos de la Organización Mundial de la Salud sobre el futuro del médico de familia, en otras palabras, el futuro de la medicina general. Vino a Chapel Hill y estábamos en el bar del hotel Carolina Inn tomando una copa antes de cenar. Scott preguntó: *“¿Por qué está disminuyendo tanto la práctica médica general en EEUU?*” Respondí: “*general y práctica son ahora dos palabras mal vistas en círculos académicos. Las facultades de las escuelas de medicina no quieren tener nada que ver con la preparación de generalistas. La especialización es lo que domina la actualidad. La mayoría de los miembros actuales de la facultad de medicina no parecen entender la necesidad de generalistas. Necesitamos algún otro término para este componente esencial de la atención médica*”. Scott y yo discutimos términos como “atención básica”, “atención general” y “atención fundamental”, y se me ocurrió el término “atención médica primaria” durante esa conversación. Luego, Bernie Greenberg, Frank Williams y yo incorporamos más tarde el término en el artículo de “ecología”. El famoso informe Dawson era desconocido para mí en ese momento; solo me enteré de su existencia a mediados de la década de 1970. Lord Dawson, jefe del Servicio Médico del Ejército Británico durante la Primera Guerra Mundial, había sido comisionado por su gobierno para preparar un informe sobre la futura estructura de los servicios médicos de ese país en preparación para un posible Servicio Nacional de Salud. En su informe histórico, publicado en 1920, Dawson habló de “centros de salud primarios” y presentó diagramas que mostraban cómo estos centros se irradiaban en centros de atención médica secundaria más grandes; también debía haber hospitales docentes centrales en el centro de cada complejo o región. Así que la noción de atención primaria de salud se había introducido anteriormente, aunque no lo sabía en el momento en que introdujimos un término y concepto similares.

Berkowitz: También tengo otro candidato para ti. ¿Conoces a Oscar Ewing, el administrador de la seguridad social y un defensor del seguro de salud? Tenía un gráfico similar, tal vez en el Informe Magnuson, o en uno de estos informes, que era exactamente igual. El hospital universitario en el centro y los demás hospitales a su alrededor en círculos concéntricos, así que esa idea ya existía. Howard Rusk y tipos similares de personas eran muy conscientes de este tipo de ideas.

White: Otro fue John Grant en la Fundación Rockefeller. Nació en China; creo que sus padres eran misioneros (su hijo, el fallecido Jim Grant, fue el jefe de UNICEF). John Grant había observado algo de la distribución de la atención médica en China. Uno de sus amigos cercanos y también uno de mis amigos de larga data fue el profesor “CC” Chen de Chengdu. Todavía me comunico con él dos o tres veces al año; ahora tiene más de 90 años y en su momento fue ampliamente considerado como el *señor atención médica* de China. En sus días más jóvenes, estableció el primer sistema de salud rural de China organizado sistemáticamente en Dingxian. Creo que John Grant pudo haber obtenido la idea de la regionalización a partir de su conocimiento del trabajo pionero de Chen. Desde China, John Grant se trasladó a Saskatchewan, donde ayudó al gobierno a introducir una forma de regionalización. Luego fue a Puerto Rico, donde se estaba desarrollando la emergente Universidad de Puerto Rico con su innovadora Facultad de Medicina. Ayudó a diseñar los patrones de regionalización en esa isla. Estaba la Facultad de Medicina en el centro con centros de referencia más periféricos y luego centros de atención médica primaria. Puerto Rico fue el primer lugar en América del Norte, aparte de Saskatchewan, que realmente implementó el concepto de regionalización. Así que estas ideas estaban flotando con orígenes en Gran Bretaña, China, Saskatchewan y Puerto Rico. No conozco el origen exacto de ellas. Recuerde, como mencioné anteriormente, fue John Grant de la Fundación Rockefeller quien ofreció financiar el establecimiento de una práctica grupal de profesores de escuelas de medicina universitarias prepagadas para nosotros en Carolina del Norte.

Berkowitz: Pero el término “atención primaria” es suyo, ¿verdad? Otras personas tenían estas ideas, pero no usaban esa palabra.

White: Hasta donde sé, lo originamos, ciertamente en EEUU. Dawson había utilizado el término “centro de salud primaria”, aunque sin que yo lo supiera. Pero introdujimos el término como un sustituto para la práctica médica general. En retrospectiva, creo que deberíamos haber empleado algún otro término. Ahora prefiero el concepto de “medicina general” y el término “médico generalista”. La organización actual estadounidense en el que tenemos internistas, pediatras y médicos de familia haciendo más o menos lo mismo parece bastante extraña desde el punto de vista de otros países.

Berkowitz: Cuénteme ahora sobre el artículo de “ecología”.

White: Bueno, finalizamos el artículo y lo enviamos al *New England Journal of Medicine*, y recuerdo que el editor, Joe Garland, me llamó. Estaba en una reunión en Atlantic City y tuve que responder a través de un teléfono público. Lo único a lo que se opuso en el artículo fue el título “La ecología de la atención médica”. Dije, algo en el sentido de “*pero de eso se trata todo, la palabra ‘ecología’ en el título tiene que quedarse. Acepte ese título o enviaremos el artículo a otro lugar*”. Entonces Garland aceptó amablemente nuestro título y publicó el artículo en 1961. A lo largo de las décadas, nuestros análisis se han replicado en diversos entornos y nuestros hallazgos originales han sido respaldados.

Berkowitz: La idea que extraje al observar ese gráfico es que se comienza con un embudo. Hay muchas personas en la población general, algunas de ellas enferman y muy pocas terminan en Johns Hopkins. Ese es el punto, ¿verdad? Sin embargo, Johns Hopkins está formando personas solo para ver a las personas que están en Johns Hopkins. ¿Tengo la idea correcta?

White: Sí, absolutamente. Recuerdo una conversación en 1975 o 1976 con Victor McKusick, un distinguido profesor de medicina en Hopkins y en ese momento presidente del Departamento de Medicina. Le dije: “*Tenemos un terrible problema con la distribución de especialistas en este país y también con la mezcla de generalistas y especialistas*”. “*Sí*”, respondió, “*es simplemente terrible y está empeorando*”. *“¿Qué va a hacer Hopkins al respecto?*”, pregunté. “*Nada*”, respondió. “*¿Por qué no?*”, fue mi respuesta. “*Porque todos nuestros especialistas consiguen buenos trabajos*”, dijo. Esa es la visión del mundo médico de la escuela de Hopkins.

Berkowitz: Ese artículo se publicó en 1961 y realmente se convirtió en una de sus piezas distintivas.

White: Aparentemente, sí. No me di cuenta en ese momento, pero tuvo aceptación. Por supuesto, inicialmente recibimos algunas cartas con bastante enojo de diversas personas que querían saber: “*¿De qué trata todo este ‹material›? En los centros de enseñanza es donde tiene lugar la ‘verdadera’ acción médica y deberíamos centrarnos en las necesidades de los pacientes gravemente enfermos*”. Pero, como dije anteriormente, nuestros estudios han sido replicados en muchas situaciones y las relaciones básicas parecen mantenerse. En 1973, escribí el artículo principal para una edición de *Scientific American* dedicada a la atención médica. Mi contribución titulada “*Life and Death and Medicine*” utilizó cifras anuales, en lugar de mensuales, del Centro Nacional de Estadísticas de Salud de EEUU, que continuaron respaldando las relaciones en el artículo original de “ecología”. Los ilustradores de *Scientific American* dibujaron los cuadros en tres dimensiones y se han copiado con frecuencia.

Berkowitz: ¿Está cambiando eso ahora? Por ejemplo, me sometí a una tiroidectomía hace un par de años que se realizó en Hopkins. No fue algo particularmente sofisticado, pero se hizo en Hopkins, y parecían realmente ansiosos de que fuera. Estaban ávidos de que la gente fuera a esas operaciones rutinarias.

White: A principios de la década de 1960, un amigo mío, Don Anderson, que anteriormente había estado en la Universidad de Columbia Británica como profesor de epidemiología, realizó un estudio de patrones de derivación del principal hospital docente de Vancouver. Hizo que todos los especialistas del hospital, el principal centro de atención terciaria para esa provincia, indicaran qué pacientes necesitaban absolutamente ver a un especialista hospitalario. Cuando se disipó el humo, parecía que entre 100 y ciertamente no más de 200 camas por millón de población podrían ocuparse de toda la atención terciaria de esa provincia. Entonces, la respuesta corta es que estos grandes hospitales docentes con exceso de camas efectivamente tienen hambre de pacientes.

Berkowitz: Mi caso ciertamente no era una situación de atención terciaria. Era algo que podría haber sido realizado por casi cualquier cirujano.

White: Aquí es donde entran en juego la atención primaria y los llamados “guardianes de acceso”.

Berkowitz: Se me argumentó que si hubiera sido maligno, ¿no me gustaría acudir a la clínica de tumores? Él esperaba que yo pasara a la siguiente etapa, en cuyo caso sería atención terciaria. Pero, como la mayoría de estos casos, no lo era; era perfectamente normal.

White: Ellos dicen: “*estarías en manos excelentes aquí; podrían ocurrir cosas terribles allá afuera si dejas que alguien más lo haga*”.

Berkowitz: Es interesante. Permíteme hacerte otra pregunta en particular. Dijiste que cuando estabas hablando con el editor sobre el artículo de “ecología” estabas en Atlantic City. ¿Qué reunión era esa?

White: Solían ser conocidas como las “reuniones de Atlantic City” e incluían a la Sociedad Estadounidense para la Investigación Clínica, la Federación Estadounidense para la Investigación Clínica, la Asociación de Médicos Estadounidenses y quizás un par de otras organizaciones de investigación médica menos relevantes. Las reuniones colectivas duraban cuatro o cinco días.

Berkowitz: ¿Y este era el lugar al que iban los médicos académicos?

White: Sí. Este era el punto culminante intelectual del año para los académicos investigadores.

Berkowitz: Tuve una entrevista en otro proyecto con Robert Glaser.

White: Ah, sí, conozco a Bob Glaser.

Berkowitz: Es un tipo realmente político. Habló de lo buenas que eran esas reuniones en Atlantic City. Dijo que tus reuniones eran esas reuniones. No ibas a la Asociación Médica Estadounidense ni a la Asociación Estadounidense de Salud Pública.

White: Sí, bueno, más tarde asistí a algunas de esas reuniones y ofrecí charlas en encuentros tanto de la American Medical Association como de la American Public Health Association. Estuve en el consejo de gobierno de esta última durante varios años y participé en su sección de atención médica durante un número de años. Me desencantó su falta de influencia en las políticas públicas y su desinterés en la investigación sobre la adecuación de los servicios de salud. Promulgué lo que he llamado la “regla de White”, a saber, “que la influencia de cualquier organización es inversamente proporcional al número de resoluciones que aprueba”. La American Public Health Association solía aprobar resoluciones como: “*Dejarás de fumar*”, “*Harás esto y no harás aquello*”, pero no cambiaban nada. Me parecía que en la clínica se encontraba la acción y el poder, y que necesitabas involucrar a médicos y médicas para fomentar cambios significativos en el comportamiento y el estilo de vida; todas esas declaraciones y resoluciones no lograban nada. También estuve en varios comités de la American Medical Association de manera intermitente. Tampoco eran demasiado efectivos. Debo enfatizar que la investigación y el pensamiento de Jerry Morris, a quien mencioné anteriormente, me atrajeron enormemente. En 1959 solicité con éxito una beca del Commonwealth Fund para Inglaterra, donde pasé un año altamente provechoso principalmente con Morris, pero también asistiendo a la London School of Hygiene & Tropical Medicine, tomando cursos de epidemiología y estadísticas de Bradford Hill, Donald Reid y otros. Bradford Hill era el decano y desarrolló el diseño inicial para el ensayo clínico aleatorizado, que como probablemente sabes, es el estándar de oro para el énfasis actual en la medicina basada en la evidencia. Richard Doll también formaba parte del personal en ese momento como instructor, ahora es Sir Richard Doll, un destacado epidemiólogo con reputación global. También visité a varios otros líderes en epidemiología británica y actividades relacionadas, incluidos Archie Cochrane de Cardiff, Roy Acheson y Walter Holland de Londres; Tom McKeown de Birmingham; Bob Logan, Douglas Black y Robert Platt de Manchester; John Pemberton de Belfast, Donald Acheson de Oxford, Douglas Baird y su distinguida esposa de Aberdeen; y John Brotherston de Edimburgo. Todos, excepto Acheson, Holland, Logan y Black, han fallecido desde entonces. También visité a varios médicos de atención primaria prominentes, incluidos John y Elizabeth Horder, John Fry, Stuart Carne, Phillip Hopkins y John Hunt (más tarde Lord Hunt, fundador del Royal College of General Practitioners), todos de Londres; Tev Eimerl de Manchester; Richard Scott de Edimburgo, y muchos otros. Me hice amigo de Michael y Enid Balint y asistí a varias de las renombradas sesiones clínicas de esta última con médicos generales. Hubo muchas otras visitas, todas ellas relatadas en mi informe bastante completo al Commonwealth Fund al finalizar mi año sabático. Aprendí mucho de todos ellos. ¿Has oído hablar de la Colaboración Cochrane?

Berkowitz: No.

White: Visité a Archie Cochrane. Era un escocés irónico con un glorioso sentido del humor. Recuerdo haberlo visitado en Cardiff. Me preguntó acerca de mis actividades y le respondí que estaba interesado en aplicar métodos epidemiológicos al estudio de lo que en aquellos días llamábamos atención médica, pero que ahora denominamos “servicios de salud”. Pensó que era una mala idea y que la epidemiología debería tener un enfoque bastante estrecho en enfermedades infecciosas, pero podría aplicarse al estudio de enfermedades crónicas. La aplicación de conceptos y métodos epidemiológicos al estudio de los servicios de salud era, en la opinión de Cochrane en ese momento, inapropiada. Los epidemiólogos británicos enfatizaban la investigación de enfermedades crónicas a pesar de que los epidemiólogos de enfermedades infecciosas, en su mayoría estadounidenses y de Europa del Este, no estaban dispuestos a aceptar tales distorsiones de la “disciplina” inicialmente. Aprendí de Archie Cochrane el valor de estudiar poblaciones relativamente grandes mediante muestras cuidadosamente seleccionadas y la importancia de mantener el contacto con ellas para encuestas repetidas a lo largo de los años. También enfatizó la importancia de los ensayos clínicos aleatorizados doble ciego y de estudiar la distribución de los llamados fenómenos “normales”. Por ejemplo, los estándares de referencia utilizados en los laboratorios de la época proporcionaban valores “normales” para varios elementos sanguíneos para establecer la presencia de anemias clínicas. Lamentablemente, esos “estándares normales” se basaban en muestras obtenidas de personas voluntarias encontradas en centros médicos, a menudo pacientes enfermos. A veces esas personas voluntarias formaban parte del personal o eran estudiantes o pacientes “sanos”. Los estándares no se derivaban de una base de población general grande. Archie y uno de sus colegas realizaron estudios de base poblacional de la distribución de los niveles de hemoglobina y de los diferentes tipos de anemia, por lo que la profesión médica tenía un conocimiento más preciso y realista a qué denominar “normal”. Este es el tipo de cosas que John Ryle de Oxford abordó en su ensayo sobre la pregunta: “¿Qué es normal?” que mencioné anteriormente. Aprendí mucho de Archie Cochrane. Debería continuar con esta historia porque más tarde Cochrane comenzó a examinar los servicios de salud y escribió un pequeño volumen clásico, *Efectividad y eficiencia: Reflexiones aleatorias sobre los servicios de salud*, publicado en 1971 por el Nuffield Provincial Hospitals Trust. Atrajo mucha atención en ese momento, pero el mensaje fundamental no fue fácilmente aceptado por la profesión médica. Cochrane planteó la urgencia de examinar cuidadosamente las pruebas que respaldan la decisión de que cada intervención médica hace más bien que mal y citó numerosos ejemplos en sentido contrario. Por ejemplo, mostró que el consumo de vitamina B-12 en Gran Bretaña era algo así como 8 o 9 veces la cantidad requerida para tratar todos los casos en ese país de anemia perniciosa, para la cual es específica. El resto de su uso simplemente actuaba como un placebo costoso. Cochrane citó a un observador de la escena que comentó que los servicios de salud le recordaban a un crematorio: ¡entra tanto y sale tan poco! Me impresionó mucho el pequeño volumen de Cochrane; fue una presentación concisa de algunos conceptos extremadamente importantes que afectaban la práctica médica. En 1959 había conocido a Gordon McLachlan, “el secretario”, como le decían, aunque en realidad era el presidente del Nuffield Provincial Hospitals Trust. McLachlan había encargado y publicado el pequeño tratado de Cochrane. Acordé con Gordon obtener unas 200 copias de *Efectividad y eficiencia* (podrían haber sido considerablemente más) para su distribución gratuita en la reunión de 1974 del Instituto de Medicina. Y así fue como lanzamos en este país tanto a Archie Cochrane, como a muchos de los aspectos de la preocupación ahora en auge por la calidad de la atención médica. A principios de la década de 1990, Iain Chalmers y sus colegas en Oxford establecieron lo que se conoce como la Colaboración Cochrane. Ahora hay unidades en todo el mundo que buscan en la literatura médica artículos que describan ensayos clínicos para ver qué formas de intervención están respaldadas por pruebas que, de hecho, sean más eficaces que otras. Están acumulando enormes bases de datos y comenzando a tener un gran impacto en la práctica de la medicina. Inicialmente le había sugerido a Iain Chalmers que deberían hacer como McDonald’s y franquiciar los Centros Colaboradores Cochrane en todo el mundo, y eso es esencialmente lo que han hecho. Así que esa es la Colaboración Cochrane global. La profesora Kay Dickerson en la University of Maryland es el punto de anclaje clave en este país.

Berkowitz: Resulta interesante que no hable mucho de economía. Teniendo formación en esta disciplina, lo que está describiendo se asemeja a un estudio de costo-beneficio en términos económicos.

White: Me encontré con eso también en Gran Bretaña. El primer estudio de costo-beneficio con el que me topé, uno de los primeros que se hizo, fue para la línea Victoria del metro de Londres. Ahí es donde se introdujeron muchos de los conceptos y métodos relacionados con este tema. Sí, la economía de la medicina y los servicios de salud siempre me han interesado. Por supuesto, el Servicio Nacional de Salud británico me fascinó desde sus primeros días. Tengo copias del informe Beveridge original y del primer Libro Blanco del Gobierno británico que establece los objetivos del nuevo Servicio Nacional de Salud, ambos publicados a principios de la década de 1940 antes de que terminara la Segunda Guerra Mundial. En la década de 1960, el National Health Service (NHS) ya tenía 12 años y en este país todavía estábamos debatiendo sobre qué debería hacerse para aumentar la disponibilidad de la atención médica.

Berkowitz: Todavía están debatiendo en este país sobre qué podría hacerse.

White: Oh, sí. De hecho, acabo de leer en *The Economist*, mi revista de noticias favorita por casi setenta años, un extenso artículo sobre atención médica. Actualmente hay un caos total en este país. Otra contribución de los británicos al campo emergente de la investigación de servicios de salud fue el establecimiento de su primera revista científica. Abraham Marcus, también un amigo mío, a quien conocí por primera vez en 1959, era el reportero médico de *The Observer* de Londres. Comenzó la revista *Medical Care* en 1962. Pitman era el editor, pero alrededor de 1965 decidieron que el mercado no era lo suficientemente grande como para mantener una revista de ese tipo. Yo estaba en su junta editorial original y negocié la transferencia de los derechos de publicación de Pitman, el editor británico, a Lippincott en este país. La sección de *Medical Care* de la *American Public Health Association* asumió la responsabilidad de la revista. Me pidieron que asumiera como editor, pero tenía las manos llenas con otras responsabilidades en Johns Hopkins.

Berkowitz: Entonces, ¿qué se publica en *Medical Care*? ¿Quién publica, médicos académicos o epidemiólogos?

White: Los epidemiólogos pueden o no ser también médicos académicos. Los llamados investigadores de servicios de salud, algunos de los cuales son epidemiólogos, son los principales contribuyentes. Es principalmente una revista de investigación, no tanto descriptiva. La mayoría de los artículos son investigaciones revisadas por pares. *Medical Care* acaba de publicar un suplemento especial sobre la reducción del personal hospitalario, especialmente reduciendo la proporción de enfermeras registradas con respecto a los pacientes y la sustitución de “ayudantes” con formación mínima y a menudo sin licencia. Escribí la introducción y Linda Aitken está dirigiendo este gran estudio internacional desde su centro de investigación en la University of Pennsylvania. Uno de los primeros artículos publicados en *Medical Care* fue sobre los hábitos y patrones de prescripción de los médicos que atienden en una ciudad del noreste de Inglaterra. Para resumir una larga historia, encontraron que solo alrededor del 10% de las recetas eran definitivamente específicas para los fines para los que habían sido recetados. Otro 15 o 20% probablemente eran útiles, y aproximadamente la mitad de ellos eran de valor completamente dudoso. Me pareció que estas observaciones clamaban por una investigación más a fondo. Tomé esa cifra del 10% y la difundí en varios artículos y charlas. Cuando estuve en el comité asesor de la Oficina de Evaluación de Tecnología del Congreso, esa cifra fue inmortalizada en gran parte de su literatura y citada en muchos lugares. En 1976, Archie Cochrane y yo estábamos haciendo una especie de espectáculo de perros y ponis en Nueva Zelanda. En un evento conjunto en el Hospital Wellington, la audiencia estaba compuesta en gran parte por clínicos *pukka* [formales] con bata blanca y formación británica, y no quería asustarlos demasiado, así que dije: “*Solo algo en el orden del 15% o posiblemente el 20% de todas las intervenciones de los médicos tienen alguna evidencia objetiva de que hacen más bien que mal*”. Cochrane, en medio de mi frase, me interrumpió diciendo: “*Kerr, eres un maldito mentiroso. Sabes perfectamente que no es más del 10%*”.

Berkowitz: ¿Qué haces con información como esa? Entonces puedes demostrar que las intervenciones no siempre son efectivas, o en promedio no son efectivas. ¿Y qué? ¿Qué te da eso?

White: Bueno, tomemos a estudiantes nuestros en Johns Hopkins como Jack Wennberg y Bob Brook. Recuperemos primero a Wennberg. Jerry Morris me presentó el trabajo de un médico inglés llamado Glover. En la década de 1930, Glover publicó una serie de artículos que informaban sobre grandes diferencias en las tasas de amigdalectomía para niños en ciudades provinciales catedralicias británicas comparables. Pensé que estos hallazgos exigían un escrutinio más cercano. ¿Cómo es que se dan todas estas diferencias? O los niños están más enfermos o los médicos son diferentes en las diversas ciudades estudiadas. Había algo extraño sucediendo. Esto fue antes de que existiera un Servicio Nacional de Salud en el Reino Unido, por lo que tal vez podría explicarse por diferencias en el acceso a la atención. Estos hallazgos se conocieron como el “fenómeno Glover”. Cuando regresé a la University of North Carolina, le sugerí a la escuela de medicina que examináramos los datos de altas de nuestro hospital utilizando un sistema ideado por Virgil Slee en la organización del Sistema de Actividades Profesionales en Michigan, que había sido respaldado por la Fundación Kellogg. Desafortunadamente, sus resúmenes tenían demasiados datos y no suficiente información, pero era realmente el único sistema disponible en ese momento. Sugerí que intentáramos instalarlo en Chapel Hill. Se rieron de mí. Mis colegas dijeron: “*Obviamente no sabes dónde estás. Este es un centro académico universitario de enseñanza. Somos el estándar de oro para la atención médica; cuando decimos que es así, es porque es así. No necesitamos todo este tipo de cosas*”. Respondí: “*Bueno, si es así, entonces soy como Alicia en el país de las maravillas, preferiría verlo hecho en papel*”. Pero no me dejaron. Así que nos embarcamos en un estudio análogo, aunque más sencillo, de resúmenes de clínica médica general ambulatoria de nuestro hospital. Pensamos que conducíamos un barco bastante ajustado allí y que estábamos brindando lo que considerábamos una atención ejemplar y que estábamos llevando registros cuidadosos. Cuando examinamos nuestros resultados, encontramos todo tipo de espacio para mejorar: resultados de laboratorio que se pasaron por alto, errores que se cometieron, cosas que no se hicieron, pacientes que no cumplieron con las citas con sus médicos remitentes o que no regresaron a nuestra clínica según lo programado. Publicamos nuestros hallazgos en lo que entonces era el *Journal of Chronic Disease*, y que ahora es el *Journal of Clinical Epidemiology*. Bob Huntley fue el autor principal. Creo que el nuestro fue el primer artículo sobre la calidad de la atención en un entorno ambulatorio. Me interesé cada vez más en este problema y me pregunté cuáles serían los hallazgos en un estudio de base amplia en una población general. Cuando se me ofreció un trabajo en la University of Vermont en 1962 como jefe de lo que creo que fue el primer departamento de una escuela de medicina que utilizó el término “epidemiología”, lo vi como una oportunidad para realizar investigaciones basadas en la población en un estado relativamente pequeño. Lo llamamos Departamento de Epidemiología y Medicina Comunitaria. Yale, a principios de la década de 1960, se había referido a su Escuela de Epidemiología y Salud Pública, que a su vez era parte administrativamente de la Escuela de Medicina, pero era esencialmente una escuela de salud pública.

Berkowitz: Ese departamento en Yale era bastante distinguido. Muchas personas habían estado allí, por ejemplo, aquellas personas en el comité sobre costos en atención médica. Falk había estado allí.

White: Sí, absolutamente. Era un buen amigo mío. No estoy seguro de que siempre recibiera el crédito que merecía.

Berkowitz: Probablemente era una persona muy abrasiva. Nunca lo he conocido.

White: Sí, lo era en cierta medida. Recuerdo que estuve involucrado en una visita del National Institutes of Health (NIH) para una subvención con él una vez, y no manejó muy bien la visita con los representantes de la sección de estudio. Causó cierta fricción con algunas personas y creo que recibió un trato bastante duro.

Berkowitz: ¿El suyo fue el primer Departamento de Epidemiología y Medicina Comunitaria?

White: Hasta donde yo sé, sí. De todos modos, me atrajo un estado pequeño con una población relativamente estable y la perspectiva de instalar un sistema de datos de alta hospitalaria de base poblacional en todo el estado. Así que cuando llegué allí, procedimos a hacer precisamente eso. La universidad me dio un gran presupuesto y también recibí una gran subvención federal del NIH para lograr mi objetivo. Resultó ser una tarea mucho más difícil de lo que había previsto. Tuvimos bastantes problemas. La sociedad médica me llamó comunista y fui llevado ante los directivos de la sociedad médica. Hablé en salas llenas de abogados, juntas hospitalarias y administradores. Pero finalmente instalamos el Sistema de Actividades Profesionales de Virgil Slee en todos los hospitales de Vermont. Tuve varias discusiones largas con Virgil Slee, el fundador de este sistema para moteles y otros lugares variados. Cuando fui a Hopkins, Jack Wennberg fue uno de nuestros primeros estudiantes. Al graduarse, estaba buscando trabajo. Le dije: *“¿Por qué no te llevamos a Vermont y puedes examinar los datos de resumen de alta hospitalaria para todo el estado?*”. Wennberg aceptó el trabajo en Vermont y comenzó a analizar los datos. Examinó las distribuciones y variaciones en las tasas de intervenciones y procedimientos, particularmente en pacientes de Medicare, en diferentes jurisdicciones geopolíticas. Él y Alan Gittelson, un estadístico en Hopkins, eran amigos y trabajaron juntos en los estudios iniciales. Luego, Wennberg examinó datos de Maine y New Hampshire. Ahora es una autoridad internacional en estos asuntos y dirige un innovador Centro de Ciencias Clínicas Evaluativas en Dartmouth College. Lamentablemente, nunca se refieren a Glover como el que dio origen a los conceptos básicos que fueron aplicados de manera tan elegante. Escuchar a Jack contarlo da la impresión de que toda la idea surgió con él. Creo que es importante dar crédito a la persona y al lugar donde surgió. La observación inicial de que hay grandes variaciones geográficas en una amplia variedad de maniobras clínicas provino de Glover, y estoy bastante seguro de que me enteré del concepto a través de Jerry Morris, en 1959, y lo traje a este continente. Nadie más, que yo sepa, lo estaba discutiendo. Durante mi año sabático en Inglaterra, también me enteré de su sistema de Análisis de Actividades Hospitalarias que introdujo George Godber, por entonces jefe médico de Gran Bretaña. Me pareció muy interesante. Recopilaron una cantidad mínima de datos sobre todas las altas hospitalarias en Gran Bretaña, pero lamentablemente no lo analizaron de manera efectiva en ese momento. De hecho, la idea de recopilar datos sobre todas las altas hospitalarias provino de Florence Nightingale, la enfermera. Observar, cuidar y contar eran sus características distintivas. Ella introdujo la idea de un resumen de alta hospitalaria modelo en la década de 1860. ¡Se tarda mucho tiempo en que una nueva idea sea aceptada por la profesión médica!

Berkowitz: ¿Alguna vez te preocupó que, al hacer toda esta investigación y tener este control sobre lo que hacen los hospitales y los médicos, puedas juzgar, en cierto sentido, la calidad de la atención? Esto lleva directamente a lo que tenemos hoy, que es la *atención médica gerenciada,* que probablemente no te gusta.

White: Bueno, me gusta y no me gusta. Estuve en la junta del primer Health Maintainance Organization (HMO) con fines de lucro, el HMO International en Los Ángeles, alrededor de 1970, y fui reprendido por mis colegas académicos que pensaban que era una idea terriblemente mala. Dije: “*sabré más al respecto después de haber estado en la junta y ver lo que sucede y aprender al respecto*”. Lo hice revisar por Bob Huntley y también por Peter Lee, el hermano de Phil Lee. Lo investigaron por mí antes de unirme a la junta.

Berkowitz: Nunca había oído hablar de él antes, el hermano de Phil Lee, ¿era médico? Vaya familia.

White: Sí. Oh, su padre también era una gran persona. Fundó la Clínica Palo Alto y, entre otras importantes contribuciones, instó a este país a adoptar un seguro de salud nacional. Peter realizó un estudio sobre las iniciativas de subvenciones del Commonwealth Fund para implementar lo que su personal llamaba “atención integral”. El fondo fue la principal fuente de apoyo para nuestros programas educativos e investigativos en la clínica general de la University of North Carolina que mencioné anteriormente. Nuestras actividades se discutieron con cierta extensión en el volumen de Peter para el Commonwealth Fund. Volviendo a mi incorporación a la junta de HMO International, también pedí a un profesor de medicina de Stanford que evaluara la calidad de la atención brindada por HMO International antes de aceptar unirme a su junta, para poder tener una idea bastante buena de toda la empresa. También pedí a Nathan Stark que se uniera a mí en la junta como uno de los dos directores externos y él aceptó. Nathan había sido vicepresidente de Hallmark Cards y fue en gran medida responsable del desarrollo del Truman Medical Center en Kansas City. Se convirtió en vicerrector de Asuntos de Salud en la University of Pittsburgh y más tarde en Subsecretario de Salud, Educación y Bienestar en el Gobierno Federal. Debo decir que ambos aprendimos mucho al estar en esa junta. Nos encontramos con algunos socios bastante codiciosos que dirigían la empresa, pero la mayoría de la atención clínica era bastante buena. Entre otras medidas para mejorar la calidad de los servicios, estaban introduciendo sistemas de información sensatos y oportunos para monitorear la atención. Finalmente, la empresa fue comprada por la empresa Cigna y se convirtió en el núcleo del sistema de atención médica de Cigna. En general, aprendí mucho sobre diversos aspectos de los HMO y la llamada “atención médica gerenciada” en sus primeras etapas. Eso fue hace más de 25 años. En 1967, di una charla en la reunión anual de la American Association for the Advancement of Science en New York. Gerry Piel, el editor de *Scientific American*, me pidió que lo hiciera. Entre otras cosas, sugerí que EEUU terminaría con una “cuarta parte” involucrada en la organización de los servicios de salud. Habría un “comprador”, ya sea un individuo (como paciente o paciente potencial) o una organización en nombre de sus empleados o miembros, como la primera parte; el llamado “proveedor” de atención como la segunda parte; el “asegurador” como la tercera parte; y el “gestor” como la cuarta parte. También dije que este país eventualmente experimentaría una consolidación e integración vertical de la atención y los servicios. Estas estructuras organizativas emergentes no incluirían un servicio nacional de salud como en Gran Bretaña, pero probablemente terminarían con algo del orden de seis u ocho organizaciones o sistemas nacionales de salud.

Berkowitz: Es como lo que propuso Clinton.

White: Algo así, pero las entidades que imaginé serían enormes. Seguiríamos el modelo de las aerolíneas en su consolidación gradual desde aerolíneas locales y “*barnstormers*” hasta aerolíneas regionales y sistemas nacionales cada vez más grandes. Eventualmente podrías tener opciones, por ejemplo, de American, United o Continental Health Care Systems y dentro de eso tendrías una elección de clínicas y dentro de ellas una elección de médicos. Probablemente no tendrías opciones infinitas. Sobre todo, los sistemas estarían gestionados de manera eficiente y efectiva para que hubiera un servicio sin fisuras desde la atención domiciliaria hasta la atención primaria, la atención secundaria en hospitales comunitarios y la atención terciaria en los centros médicos más grandes. Aquí es donde entra mi formación e interés en la economía de la salud. Por lo que he aprendido sobre los hospitales durante décadas, muchos están pésimamente gestionados. Todavía lo creo, incluso nuestro hospital universitario local. Sus prácticas de gestión son atroces. Hay un enorme desperdicio, comunicación y organización ineptas y baja moral del personal. La industria ha conocido estos principios gerenciales elementales durante sesenta años o más. *The Economist* dice que ahora hay alrededor de 1.000 HMO y que ven que se reducirán en una o dos décadas a unas 30. Mi cifra era de unas seis u ocho. No sé si son seis, ocho o 30, pero mi suposición es que esa es la dirección en la que irá la consolidación. En mi charla en la American Association for the Advancement of Science (AAAS) sugerí que algunas de estos megasistemas serían propiedad de compañías de seguros, algunos serían propiedad de sindicatos laborales, algunos serían propiedad del gobierno, algunos de entidades sin fines de lucro y otros posiblemente de corporaciones con fines de lucro. También recomendé que se establecieran dos organizaciones, una equivalente a la antigua Civilian Aviation Board (CAB) para asegurar que hubiera una cobertura adecuada. Si vas a recibir una franquicia en Baltimore, también tendrás que proporcionar servicios en las áreas rurales de Maryland. En segundo lugar, recomendé que se estableciera una entidad equivalente a la Federal Aviation Administration (FAA) para monitorear la calidad de la atención. Así que no estaba demasiado alejado de la iniciativa de Clinton. Sin embargo, su esfuerzo fue completamente erróneo. Clinton debería haber dado al Congreso cuatro o cinco principios importantes relacionados con la prestación de servicios de salud y luego dejar que elaboraran las formas, medios y leyes. Los principios podrían incluir: cobertura universal, portabilidad en todo el país, cobertura integral, que sea gratuita en el punto de servicio y pagada a través de una combinación de primas individuales, contribuciones del empleador e impuestos generales estatales y federales. Eso es lo que sucedió con la Comisión Hall en Canadá. Varios de mis antiguos alumnos participaron en el esfuerzo de Clinton, así que tuve una idea de lo que estaban haciendo. Pero sobre la idea de HMO. Paul Elwood y yo estábamos en uno de los Foros de Sun Valley patrocinados en parte por el Commonwealth Fund en la década de 1970. El tema general, según lo recuerdo, era la organización eficiente de la atención médica. Presenté un artículo que le gustó. Aparentemente eran ideas nuevas para él. Expuse muchos de los conceptos expresados en el artículo de la AAAS que describí anteriormente. Esbocé una disposición jerárquica y la necesidad urgente de gestionar los servicios de manera más efectiva y eficiente y de proporcionar servicios sin fisuras con respecto a registros, comunicaciones y la derivación del paciente entre diferentes niveles de atención y centros. Le gustaron las ideas. Unas semanas después tuvimos una conversación telefónica de dos o tres horas un sábado por la tarde. Estaba discutiendo la idea de poner en marcha la noción de HMO o *health maintenance organization*. No generé el término; él generó el término, pero el concepto, creo, vino de este Foro de Sun Valley. Para dar crédito donde se debe, incluso mis ideas se basaron, a su vez, en la experiencia de Kaiser. Fue el primer HMO a gran escala.

Berkowitz: El Group Health en Washington es más antiguo. Has hablado sobre el efecto placebo en relación con tu trabajo. ¿Tienes una opinión al respecto? ¿Conoces el libro *Persuasion and Healing* de Jerome Frank, quien fue tu colega en Hopkins? Él habla sobre el efecto placebo. Su idea es que no es tan malo. Si funciona, está bien. Si las personas toman una píldora de azúcar y se sienten mejor, no deberíamos desestimarlo. Tú no lo haces, ¿verdad?

White: Creo que es la intervención terapéutica ubicua más subestimada que tenemos. Tiene un efecto extremadamente poderoso y de propósito general, y es “el cuidado”. Es una manifestación del amor, si quieres.

Berkowitz: Entonces, ¿no te opones a eso?

White: ¿Oponerme? Estoy muy a favor. De hecho, le escribí a John Eisenberg, quien es un viejo amigo mío, cuando se convirtió en administrador de la Agency for Health Care Policy and Research. Cliff Gaus, un antiguo estudiante de mi departamento en Hopkins, había sido el jefe anterior. Cuando Cliff regresó a la vida civil, John Eisenberg tomó el relevo. Le escribí y le dije: “*Hay dos cosas que creo que necesitas examinar. Una es el efecto placebo y la otra es la actual reducción del personal de enfermeras registradas y su reemplazo por ‘auxiliares’ con formación mínima, sin experiencia, mal formadas y sin licencia*”. Es una forma escandalosa de “ahorrar dinero”. El efecto placebo necesita ser examinado mucho más. Cada vez hay más literatura al respecto. Un profesor de Gastroenterología en Yale, Howard Spiro, escribió un libro interesante sobre el efecto placebo. Mostró que había grandes diferencias entre Londres y Dundee en las tasas de curación y el efecto placebo para regímenes de tratamiento idénticos para la úlcera péptica, aunque se utilizó el mismo protocolo en ambos lugares. También se encontraron grandes diferencias entre Inglaterra y EEUU en las tasas de curación y placebo para la enfermedad de úlcera péptica, aunque nuevamente estaban usando las mismas píldoras y los mismos protocolos. Claramente, había algo más además del régimen terapéutico y el efecto placebo. Probablemente era el efecto Hawthorne que, a su vez, está relacionado con la cultura general de una organización, el clima gerencial en el que opera el hospital, incluidas las actitudes administrativas hacia los empleados y las enfermeras, y la reputación general del hospital y sus médicos. De alguna manera, esto se transfiere de modo que influye en las tasas generales de curación de toda la institución. Hace más de treinta años, el profesor Reginald Revans de la University of Manchester, respaldado por el Nuffield Provincial Hospitals Trust, mostró esto en un conjunto clásico de estudios en diferentes hospitales y para diferentes condiciones. Cuanto más autoritaria era la administración de hospitales de tamaño similar, mayor era la rotación laboral de enfermeras y otros empleados y más larga era la estancia hospitalaria del paciente para seis afecciones médicas comunes y seis afecciones quirúrgicas comunes. Lo contrario, por supuesto, también era cierto. 

B**erkowitz:** Aunque eso es bastante difícil de replicar, ¿verdad? El objetivo de todo esto es replicar, ¿correcto? La única razón para hacer toda esta investigación es que estás intentando generalizar y luego replicar en otros lugares, ¿verdad? Tal vez es solo que un médico es mejor, que un médico tiene un mejor tacto que otro, lo cual puede no ser replicable más que una escuela modelo es replicable en otro lugar. Podría depender simplemente de un profesor. Hablamos del efecto Hawthorne de una manera generalizada, pero podría haber sido particular para esa sede de Western Electric y esos experimentadores de la Harvard Business School o de donde fueran, que eran cálidos con ellos, que se sentían queridos.

White: Creo que esto debe ser examinado; el fenómeno debe estudiarse mucho más en entornos de atención médica. Por supuesto, es ampliamente aceptado en la industria, el comercio y otros entornos organizacionales. Los hospitales parecen ignorarlo. Deberíamos analizar los diversos componentes de los efectos placebo y Hawthorne para determinar cómo influyen en las respuestas inmunitarias, y otras respuestas humorales y endocrinas, y neurotransmisores, tanto en el médico como en el paciente. Algo de esto se ha hecho en varios lugares. No sé si estás familiarizado con la revista trimestral *Advances* publicada por el Fetzer Institute en Michigan. Muchos de estos temas se discuten con cierta extensión. Necesita hacerse mucha, mucha más investigación sobre ellos.

Berkowitz: Escucho una visión muy optimista de que la ciencia puede triunfar aquí, de que realmente podemos entender esto. Algunas personas dirían que realmente no entendemos esto. Un tipo es un buen médico rural, no sabemos por qué es bueno, y nunca vamos a saber por qué. Es utópico pensar que vamos a poder entender la química cerebral que explica por qué las personas son receptivas a alguien. Estás bastante optimista al respecto.

White: Oh, sí. Necesita mucho trabajo, pero es un área legítima para la investigación. De hecho, Larry Green, un protegido mío que es jefe del Departamento de Medicina Familiar en la University of Colorado, recibió el Premio Curtis Hames este año. Me envió una consulta diciendo: “*¿Qué opinas sobre la investigación en medicina familiar?*” Le dije: “*No pienso mucho en ello. Creo que realmente están perdiendo la oportunidad*”. En lugar de hablar sobre la falta de asistencia a citas, la carga de pacientes, los programas de residencia y muchos fenómenos efímeros, realmente deberían estar observando la historia natural de la enfermedad y qué es lo que hace que ciertos médicos logren curas. Deberían estar estudiando los orígenes y la historia natural de enfermedades como la insuficiencia cardíaca o la úlcera péptica. Ahora están en esta gran tendencia de antibióticos para la úlcera péptica porque encontraron una bacteria que está allí. Bueno, hay bacterias flotando por todas partes, pero cualquiera que haya hablado con un paciente con úlcera péptica sabe que también están bajo un gran estrés y tensión cuando la úlcera se vuelve sintomática. Entonces no es una cosa u otra. Es una combinación de factores. Hay una red de causalidad. Esto necesita ser desentrañado. Hay toda un área de investigación aquí que mejor se puede hacer en entornos de atención primaria pero que, en su mayor parte, está completamente descuidada.

Berkowitz: Entiendo.

White: Regresemos a Vermont antes de estas digresiones, volvamos a Bob Brook. En 1959, en Inglaterra, Jerry Morris había realizado estudios comparando hospitales docentes y no docentes y encontró que las tasas de letalidad para diferentes condiciones como la úlcera péptica, el infarto de miocardio y el agrandamiento de la glándula prostática variaban enormemente entre los dos tipos de hospitales. Entonces, ¿qué explicaban las variaciones?, ¿había diferencias en los patrones de referencia, diferencias en la gravedad o complejidad de los problemas de los pacientes, o diferencias en la calidad de la atención que recibían los pacientes?, ¿qué cosa única o, más probablemente, qué combinación de factores, producían las diferencias? Estos hallazgos plantearon preguntas sobre la calidad de la atención y la necesidad de examinar esto con mayor detalle. Así que también internalicé esta idea. Cuando regresé a Chapel Hill, mencioné antes que quería implementar el Sistema de Actividades Profesionales en el hospital, pero la idea fue rechazada. Realizamos estudios de calidad modificados en el departamento ambulatorio. Cuando fui a Hopkins, pensé que sería bueno replicar algunas de las ideas de Morris. Bob Brook era un estudiante, así que me acerqué a Hopkins y nuevamente fuimos rechazados con la táctica: “*¡No sabes dónde estás! ¡Lo que hacemos establece el estándar de oro para la atención en todas partes!*”.

Berkowitz: Este realmente es el estándar de oro.

White: Así que fuimos con Julie Krevans, con quien trabajé bastante de cerca. Era el jefe de medicina en el Baltimore City Hospital, afiliado a Hopkins. Nos dejó hacer el estudio allí y, efectivamente, encontramos todo tipo de problemas: radiografías no revisadas, turnos cancelados, resultados de laboratorio enterrados. Bob Brook publicó los hallazgos en el *New England Journal of Medicine*. Comenzó a investigar todo este material; y ahora la “evaluación y garantía de calidad” son industrias en crecimiento. Pero estas ideas vinieron de Jerry Morris y los epidemiólogos ingleses. Los traje en 1960, habían estimulado mi interés en investigar estos asuntos. Finalmente entramos en el hospital de Hopkins propiamente dicho y encontramos los mismos problemas allí que habíamos encontrado en el Baltimore City Hospital. Se descubrieron todo tipo de desventuras allí.

Berkowitz: Volviendo a tu ida a Hopkins y dejando Vermont, ¿fue simplemente una oferta que no pudiste rechazar de Johns Hopkins para iniciar este nuevo departamento allí?

White: Sí. Aparentemente habían estado al borde de acercarse a mí antes de que me fuera a Vermont, y solo había estado allí, supongo, menos de un año antes de que me invitaran. Dije que no podía dejar la University of Vermont de inmediato. Sí, me ofrecieron un presupuesto sustancial y bienes raíces de primera, originalmente en el propio hospital. Pensé: “*Bueno, probablemente puedas decir cosas desde Hopkins que no puedes decir desde Vermont, o puedes decir el mismo tipo de cosas, pero tus colegas prestan más atención cuando vienes de Hopkins*”. Nunca se me había ocurrido antes que terminaría en Hopkins.

Berkowitz: Esta era la Escuela de Higiene, por supuesto. ¿Te preocupaba eso?

White: Sí, era la Escuela de Higiene y, sí, me preocupaba. Pero el espacio iba a estar en la Escuela de Medicina e íbamos a poder trabajar con estudiantes de medicina, así que pensé que esto estaba bastante cerca de lo que habría preferido y no se puede tener la perfección. Pero sí, me preocupaba. De hecho, había traído a John Last desde Australia para unirse a mi departamento en Vermont y lo invité a venir a Hopkins conmigo, pero él quería quedarse en una escuela de medicina. Por supuesto, ahora es el editor del ampliamente aclamado *Dictionary of Epidemiology* y del libro más vendido sobre salud pública, previo a Maxie Rosenau. Ahora está en la University of Ottawa, en una escuela de medicina. Sí, me preocupaba, y tengo que decir que, con notables excepciones, de las cuales enumeraría a personas como Abe Lillienfeld, Alan Ross y Russ Morgan en Hopkins y en la Escuela de Salud Pública de la University of North Carolina a personas como John Cassel, Al Tyroler y Bernie Greenberg, el discurso general y el nivel de actividad intelectual son considerablemente más bajos, en promedio, en las escuelas de salud pública que en las escuelas de medicina. Hay, por supuesto, algunos auténticos incompetentes en las escuelas de medicina y hay mucha actividad inútil, obviamente, pero creo que, en general, las escuelas de medicina están realmente por encima de las escuelas de salud pública, intelectualmente hablando. Pero hay algunas notables excepciones y no quiero sugerir lo contrario. He pasado aproximadamente la mitad de mi carrera en cada una de las dos culturas.

Berkowitz: Sí, sin embargo, debe haber causado mucha tensión en Hopkins, porque estoy seguro de que la gente de salud pública sentía una sensación de separación y probablemente pensaba que eso era bueno, y usted era una persona que venía diciendo: “*Lo que realmente importa es la práctica médica y tenemos que entrar allí*”.

White: Ya sabes que Medicare y Medicaid estaban empezando a entrar y creo que me contrataron para dar un curso sobre cómo llenar los formularios de Medicare. Ernest Stebbins, el decano de *Higiene*, me sugirió que debería ofrecer algo así, y dije: “*Eso no es para lo que vine aquí*” (creo que Stebbins ya está muerto). Empecé a escribir artículos y a decir cosas que pensaban que eran subversivas para los intereses de la fraternidad de salud pública. Verás, estaba el campo de la llamada administración de salud pública e inicialmente estaba vinculado a ese departamento; John Hume era el jefe. Los administradores de salud pública habían combatido la idea de involucrarse en la atención médica; estaban muy preocupados de que la AMA les hiciera cosas terribles si empezaban a meterse con la atención médica. De hecho, muchos de los actos estatales que establecían departamentos de salud dentro de los gobiernos estatales específicamente les prohibían involucrarse en la atención médica. También exigían que el comisionado de Salud del estado tuviera un Master of Public Health (MPH) otorgado por una escuela de salud pública. Mencioné a John y Elizabeth Horder y sus cajas anidadas que representaban la distribución de pacientes en su propia práctica y que había sido el modelo para nuestro propio artículo sobre la ecología de la atención médica, pero había otro médico, John Fry, un médico general de Beckingham, cerca de Londres, que murió hace unos años, que fue uno de los que dieron origen a la investigación en la práctica general. Publicó numerosos artículos basados en investigaciones en su práctica, también publicó varios libros, incluidos varios que comparaban los sistemas de atención médica en diferentes países con especial énfasis en la atención primaria. Él y un colega en la University of Manchester, Tev Eimerl, habían introducido un sistema de registro para su uso en prácticas generales. Los sencillos formularios de registro recopilaban datos básicos sobre el paciente y el motivo del contacto o encuentro con el médico general. El sistema de Eimerl se hizo famoso como el *E Book System*, nombrado en honor a su inventor. Basado en el uso de este método de registro en numerosas prácticas, el registro general del Reino Unido de asuntos médicos, W. P. D. Logan, había publicado una serie de volúmenes anuales que daban las distribuciones de problemas médicos en la práctica general. Estos fueron los primeros informes de lo que sucedía en el componente más grande de la cuestión de la atención médica: la atención primaria. Antes de eso era una “caja negra”. Hubo uno o dos estudios de prácticas individuales que se habían realizado en este país a principios de la década de 1950, incluido uno en Carolina del Norte en 1953 por el Dr. Leon Taubenhaus. Aquí estábamos, dirigiendo escuelas de medicina, sin ninguna información útil sobre la distribución de problemas de atención médica en las poblaciones atendidas. Cuando llegué a Vermont, esta fue otra idea que obtuve mientras estaba en Inglaterra. Era factible estudiar prácticas generales y el contenido y la distribución de la atención médica. Por lo tanto, introdujimos el *E Book System* en Vermont. John Last trabajó conmigo en esto y publicamos un artículo en Medical Care sobre el contenido de la práctica general. El estudio no se basó completamente en la población, pero fue un comienzo. Perseguí la idea cuando estuve involucrado con el National Center for Health Statistics. Alrededor de 1970 tuve una discusión, en realidad en el baño del Departamento de Salud, Educación y Bienestar, como se llamaba entonces, con Ted Woolsey, que era el director adjunto y luego se convirtió en director del Centro Nacional. Le dije: “*Sabes que ustedes están publicando estadísticas sobre personas que están muertas, y están haciendo un poco sobre personas que están hospitalizadas, pero no están haciendo nada sobre los problemas iniciales que se llevan a las fuentes de atención médica. Deberíamos estar estudiando los problemas de los vivos, así como recopilando datos sobre los muertos. Podríamos ser capaces de ayudar a los vivos, pero no podemos hacer mucho sobre los demás*”. Woolsey reconoció que era una buena idea y prometió hacer algo al respecto. Para hacer una larga historia corta, cuando se convirtió en director del Centro, nos encargó, alrededor de 1972, en Hopkins, desarrollar el diseño de la encuesta para la *National Ambulatory Medical Care Survey* (NAMCS) y nuestro equipo diseñó esa encuesta. Fue la primera vez que las escuelas de medicina del país habían tenido alguna información sobre la distribución de problemas de salud en la atención primaria. El National Center for Health Statistics tuvo más solicitudes para los informes de NAMCS que por cualquier otra de sus publicaciones hasta ese momento.

Berkowitz: Por supuesto, pero también hay una tradición allí, los estudios de morbilidad de Edgar Sydenstricker en la década de 1930, National Health Survey. Eran tipos de salud pública, esos chicos.

White: Sí. La operación de Sydenstricker fue el predecesor del National Center for Health Statistics. Ellos construyeron sobre su trabajo.

Berkowitz: Hagerstown, Maryland, era un lugar.

White: Pero la *National Ambulatory Medical Care Survey*, como las otras encuestas nacionales realizadas por el National Center for Health Statistics, se basaban en la población, en lugar de estudios de base poblacional con poblaciones seleccionadas o limitadas. Tuvimos bastante tiempo para conseguir que todos participaran en la encuesta NAMCS, especialmente la AMA y todas las sociedades de especialidades. Había personas que pensaban que era una trama comunista y que iban a ocurrir cosas terribles. Pero eventualmente, los informes de NAMCS se convirtieron en superventas y fueron muy demandados por los miembros de las escuelas de medicina del país y, por primera vez, se enteraron del contenido de la atención tanto en la práctica general como en la especializada. Sin embargo, los establecimientos de las escuelas de medicina que controlan el currículo no han prestado demasiada atención a los datos de NAMCS hasta hace poco tiempo.

Berkowitz: Había encuestas sobre morbilidad, había encuestas sobre discapacidad, pero ¿tu encuesta era sobre qué?, ¿qué tipo de preguntas hiciste?

White: En primer lugar, preguntamos sobre la queja, condición, problema o síntoma inicial del paciente.

Berkowitz: ¿Estas eran personas que ya estaban enfermas y que iban al médico?

White: Sí, en general, pero algunos eran personas “sanas” que iban a hacerse un “chequeo” o a buscar información. El centro nacional ya realizaba la *National Health Interview Survey* que proporcionaba información sobre lo que la gente común decía acerca de su propia salud. Queríamos averiguar qué sucedía en los consultorios médicos, qué tipo de problemas llevaban sus pacientes, cómo los llamaban, cuán urgentes eran, cuán graves eran, qué pruebas diagnósticas se realizaban, qué tratamientos o recetas se ordenaban, qué derivaciones se hacían y cuánto duraba el encuentro.

Berkowitz: Eso es interesante. ¿Y los seguiste todo el camino para ver qué les sucedía?, ¿tuviste otros médicos que miraran los casos para ver si estaban correctamente diagnosticados?, ¿eso era parte de ello?

White: No. Pero otra cosa que descubrí en Inglaterra a partir de los estudios de práctica general realizados allí fue que el 60% de los problemas llevados a las fuentes iniciales de atención médica no podían recibir etiquetas de la Clasificación Internacional de Enfermedades (CIE). Simplemente son problemas, condiciones o quejas. Tal vez el paciente no duerme bien por la noche o “*mi jefe me está acosando*”. No hay lugar para ellos en la CIE. Más recientemente, ayudé a organizar la Clasificación Internacional de Atención Primaria, publicada por Oxford University Press. Esta clasificación ahora se está utilizando ampliamente en Europa y en otros lugares, pero más lentamente en este país. Hay toda una serie de otras estadísticas de salud en las que estuve involucrado como presidente del National Committee on Vital and Health Statistics (NCVHS).

Berkowitz: ¿De quién era este comité?

White: El NCVHS era un comité asesor en asuntos estadísticos del secretario de Salud, Educación y Bienestar (Health, Education and Welfare, HEW) y el National Center for Health Statistics. Estos comités nacionales fueron originalmente establecidos por la Organización Mundial de la Salud (OMS), la cual invitó a todos sus países participantes a establecer un comité nacional sobre estadísticas de salud. Inicialmente, se iban a ocupar sobre todo de clasificar y registrar las causas de muerte y desarrollar la Clasificación Internacional de Enfermedades. Al principio era un comité voluntario hasta que conseguí que Ted Kennedy lo convirtiera en un comité estatutario por primera vez. En el mismo período, me di cuenta de que el National Center for Health Statistics tenía una serie de encuestas nacionales y sistemas de recopilación de datos no relacionados, como la *Health Interview Survey*, la *Health and Nutrition Survey*, la *Ambulatory Care Survey*, la *Hospital Discharge Survey*, la *Disability Survey* y, por supuesto, los informes de estadísticas vitales. De alguna manera, todos deberían integrarse para que el público y los políticos pudieran comprender mejor el estado de la salud de la nación y los patrones de enfermedad. Pude persuadir a Kennedy para que incluyera en la Ley de Autorización de Salud Pública una frase algo así como: “*Habrá un informe anual al Congreso sobre la salud del pueblo de EEUU*”. Cuando se publicó el primer número de *Health*, apareció en las portadas del *New York Times*, el *Washington Post* y el *Wall Street Journal*. Estaba en la oficina de Dorothy Rice esa mañana; ella acababa de asumir como directora del NCHS. Recibió una llamada de la Casa Blanca preguntando: “*¿Qué diablos es esto?*”. David Matthews era el secretario de Salud, Educación y Bienestar, durante la presidencia de Ford, 1974-1975. Por supuesto, los subalternos de Matthews habían aprobado nuestro informe, pero no habían prestado atención a lo que estaban firmando, así que Dorothy tuvo que explicar de qué se trataba. Pero ha sido una publicación muy popular desde entonces y generalmente obtiene cobertura en la primera página en la mayoría de los periódicos cada año. Solíamos hacer un libro de gráficos que los políticos usaban. Podían decir si la pendiente de la curva estaba subiendo o bajando o si las barras se estaban haciendo más grandes o más pequeñas. No podían leer una tabla estadística, pero podían mirar una imagen y obtener algún significado de ella. Eso también fue un éxito de ventas.

Berkowitz: Eso fue tu creación, ¿verdad?

White: Sí. Ayudé a armarlo. Tal vez debería mencionar que tuve varios encuentros con Kennedy a lo largo de los años. Como miembro del Comité Asesor para el Programa de Salud de la Office of Technology Assessment (OTA), a menudo tuve oportunidades de hablar con él; asistía regularmente a nuestras reuniones. En una ocasión le mencioné que el presupuesto para el National Center for Health Statistics era inadecuado para las tareas que se le exigían. Tomó nota en la parte posterior de una cubierta de cerillas. Pensé que ahí terminaría el asunto. Sin embargo, al día siguiente, estaba en una reunión en New York y me llamaron para una llamada telefónica de uno de los ayudantes de Kennedy. Quería saber más detalles precisos sobre el problema del presupuesto. Le di estos y, para resumir, Kennedy logró que se aumentara adecuadamente el presupuesto del NCHS. Su seguimiento fue bastante notable. Mientras estoy en el tema de Kennedy, recuerdo que me ofrecieron el puesto de director asociado de la Office of Technology Assessment por el entonces director, cuyo nombre olvido. Tenía que decidir entre el trabajo en la Fundación Rockefeller y el puesto en la OTA. Había rechazado este último, pero Kennedy me llamó personalmente e intentó persuadirme para que cambiara de opinión. Estuve muy tentado, pero no creí que sería feliz como burócrata. Había tenido un par de insinuaciones sobre unirme a esa fraternidad en años anteriores, pero nunca me interesaron. Sin que yo lo supiera, al parecer fui el segundo finalista como cirujano general (jefe operativo del Cuerpo Comisionado del Servicio de Salud Pública de EEUU) en la era de Johnson, pero fui vetado por el establishment de salud pública por razones obvias. Conocían mis puntos de vista sobre la formación en salud pública.

White: Cuando asumí como presidente de la Sección de Estudios de Investigación en Servicios de Salud, era en parte una entidad del NIH y en parte de la llamada fraternidad de salud pública “del centro de la ciudad”. Realmente se regía según las pautas del NIH. Había sido miembro de esa sección durante varios años antes de que me nombraran presidente. Anteriormente, esa entidad se llamaba Sección de Estudio de Instalaciones Médicas y Hospitalarias. Era la entidad que distribuía el dinero de la Ley Hill-Burton para la investigación, el primer millón de dólares para la investigación en servicios de salud alrededor de 1955. La mayoría de la investigación financiada era bastante limitada y se centraba principalmente en mejorar los hospitales. Entre las investigaciones que apoyaban incluían, por ejemplo, propuestas para reducir el ruido de los contenedores de basura, cómo reforzar el departamento de suministros centrales o cómo mejorar la comida. Era material muy básico. La investigación no tenía nada que ver con por qué se usa un hospital o si hace algún bien o no. Nuestra subvención W74 fue una notable excepción y sugirió una nueva dirección de investigación. Alrededor de 1959 hubo una reunión conjunta histórica entre otra entidad del NIH, la Sección de Estudio de Enfermería, y la Sección de Estudio de Instalaciones Médicas y Hospitalarias. Nos reunimos en el sótano del edificio Westwood, en Bethesda, en el campus del NIH. Era uno de los edificios auxiliares que estaban utilizando en ese momento, tal vez todavía lo estén utilizando. De esa reunión surgió el término Investigación en Servicios de Salud. No recuerdo haberlo hecho, pero alguien introdujo el término “investigación en servicios de salud”. Anteriormente habíamos llamado a este campo emergente “investigación en atención médica”. Eso fue cuando habíamos estado involucrados en Chapel Hill. Paul Densen, Cecil Sheps, George Reader y algunos otros, éramos un pequeño grupo que nos reuníamos periódicamente para discutir nuestra investigación. Cuando me convertí en presidente de la sección de estudio en 1962, decidí que deberíamos intentar poner nuestro nuevo campo en el mapa académico. Adoptamos los modelos que se habían utilizado para desarrollar el interés público y político en áreas hasta entonces descuidadas, como las ciencias ambientales y la genética. Esto significaba crear una comprensión del campo y hacerlo visible para una amplia variedad de posibles interesados. Así que introdujimos programas como los Premios a Jóvenes Investigadores y financiamiento para Centros de Investigación en Servicios de Salud con sede en universidades. Establecimos varios de estos en todo el país y muchos aún prosperan. Y encargamos un conjunto de documentos de académicos creíbles que revisaron las numerosas disciplinas que podrían contribuir al amplio campo de la investigación en servicios de salud. Sus documentos fueron comentados en dos conferencias importantes y todos fueron publicados por el Milbank Memorial Fund bajo la dirección de Donald Mainland, un distinguido estadístico médico y un amigo cercano mío. Toda la serie estableció por primera vez el alcance potencial, por ejemplo, de la economía, la sociología, la epidemiología y la teoría de la gestión para investigar una amplia variedad de componentes de los sistemas de servicios de salud. Se discutieron los métodos y conceptos que se utilizaron en las diversas disciplinas.

Berkowitz: ¿Esto habría sido cuándo, a principios de la década de 1960?

White: Sí. Probablemente 1963-1964, algo así. Ese fue un enfoque para establecer el campo. Hicimos varias otras cosas también. Quería enviar un equipo a Europa para observar los servicios de salud allí y el potencial para estudios comparativos de servicios de salud. Recuerdo haber hablado con la gente del Servicio de Salud Pública que decía: “*Oh, no puedes hacer eso. Allí tienen muchas ideas comunistas y socialistas. Simplemente contaminaría todo el ambiente aquí. No puedes enviar a nadie*”. Respondí: “*¿Por qué no vamos y averiguamos qué están haciendo? Si han cometido muchos errores, podríamos saberlo para evitar repetirlos. Si hay algunas cosas buenas que están haciendo, tal vez algunas de ellas puedan adaptarse para su uso en nuestro país*”. Finalmente obtuve permiso. Les aseguré: “*No estoy tratando de meterme en una gira gratuita. Yo mismo no voy a hacer este viaje*”. Convencimos a Bob Haggerty para que lo dirigiera, y más tarde me sucedió como presidente de la Sección de Estudio de Servicios de Salud. Luego, propuse que la Sección de Estudio debería visitar varios lugares que fueran influyentes en la prestación de servicios de salud o que pudieran ser lugares adecuados para la realización de investigaciones. Nuevamente, inicialmente me dijeron que esta era una mala idea; finalmente, los funcionarios de Salud Pública estuvieron de acuerdo. Con la ayuda de ese mago burocrático y consumado negociador, Tom McCarthy, el secretario ejecutivo de la Sección de Estudio, toda la Sección de Estudio visitó y celebró seminarios y conferencias extendidas con, por ejemplo, la Asociación Americana de Hospitales, el Departamento de Salud del Estado de California, los Centros para el Control de Enfermedades y el Departamento de Salud de Puerto Rico y la Universidad de Puerto Rico, donde la regionalización de los servicios de salud se estaba convirtiendo en una realidad. Luego dije: “*Queremos visitar la AMA*”. Una vez más, los tipos de salud pública nos dijeron: “*No puedes hablar con la AMA. ¡Son el enemigo!*” Esto es realmente increíble ahora.

Berkowitz: Lo eran más aún en ese momento, ¿verdad?

White: Más aún en ese momento. Dije: “*Están todos estos médicos que están haciendo todas estas cosas a la gente; necesitamos saber más sobre sus actividades*”. Y me dijeron: “*¡No puedes hacerlo!*” Tom McCarthy, a quien mencioné hace un momento y que, por cierto, deberías entrevistar porque sabe dónde están enterrados todos los cadáveres, dijo: “*Simplemente iremos y enviaremos la factura al Servicio de Salud Pública. ¿Qué van a hacer, meternos en la cárcel?*” Así fue como fuimos a Chicago y tuvimos una excelente reunión en la sede de la American Medical Association y les enviamos la factura al Servicio de Salud Pública y no pasó nada. Tuvimos conversaciones muy constructivas con ellos. Charlie Edwards fue el anfitrión; más tarde se convirtió en secretario adjunto de Salud en el Departamento Nacional de Salud, Educación y Bienestar. No recuerdo si fue en esa reunión o en una posterior, en el bar del Palmer House Hotel, cuando dije: “*Lo que necesitamos ahora que tenemos todas estas cosas en marcha, es crear un Centro Nacional para la Investigación de Servicios de Salud*”. Esbocé esto en una servilleta de papel sobre una mesa cubierta con un mantel a cuadros rojos y blancos, lo recuerdo bien. Tom McCarthy estaba allí y Evelyn Flook, uno de los burócratas que dispensaban el dinero después de que tomáramos las decisiones, y Gil Barnhart, quien tenía un papel similar. Sabíamos que la reunión anual de la Association of American Medical Colleges (AAMC) estaba en marcha en Washington DC, así que decidimos intentar distribuir un anuncio allí. Gil Barnhart y Evelyn Flook regresaron rápidamente a Washington y produjeron un folleto en unas 36 horas que describía lo que haría la entidad propuesta y justificaba su necesidad. Distribuimos cientos de copias en la reunión. Hice algunos comentarios, pero la reacción general parecía ser que yo era “*simplemente un alborotador*”. La investigación de los servicios de salud no se consideraba de interés para las escuelas de medicina del país. Su énfasis estaba en la investigación biomédica. Luego hicimos cabildeo con Phil Lee, quien era secretario adjunto de Salud en el Departamento de Salud, Educación y Bienestar, y George Silver, su subalterno, y varias otras personas. Nombraron a Paul Elwood para realizar un estudio de factibilidad; hubo varias interacciones y, finalmente, se promulgó la autorización del Centro Nacional para la Investigación de Servicios de Salud y se creó.

Berkowitz: ¿En qué año fue eso?

White: Creo que fue en 1968 cuando se creó.

Berkowitz: ¿Cuál fue su modelo?, ¿se inspiró en el modelo del NIH?

White: Creo que en el modelo del NIH, sí. Pensamos que debería haber una entidad visible y sustancial encargada de examinar tantos aspectos como se pudiera de la cuestión de servicios de salud. No estoy seguro de que hayamos detallado todos, pero ciertamente la calidad, el acceso, los costos, la eficacia y la eficiencia eran prominentes. Creíamos que debería tener un personal interno considerable, un presupuesto adecuado, otorgar subvenciones a individuos e instituciones, estimular y promover el campo, y que debería difundir los resultados. El nuevo centro podría comenzar de manera modesta, pero lo veíamos expandiéndose y desempeñando un papel esencial en la mejora de la organización de la atención médica de la nación. Una cosa que probablemente surgió en algún momento es que, si vamos a tener competencia y variación en los estilos, formatos organizativos, propiedad y patrones de personal de las instalaciones y servicios de atención médica, necesitamos poder compararlos. Necesitamos conocer mediciones de los resultados de su atención, cuáles son los costos, cuáles son los niveles de satisfacción del paciente, etc. La única forma de hacerlo es con información adecuada, su amplia difusión y discusión pública. Tienes que empezar en algún lugar. Recuerdo haber dicho que pensaba, tal vez no al principio, pero en algún momento, que realmente iba a hacer mucho ruido cuando los periódicos comenzaran a publicar comparaciones de los hospitales locales y sus tasas de mortalidad, tasas de morbilidad, costos y resultados. Efectivamente, eso es lo que finalmente sucedió. Así es como empezó.

Berkowitz: ¿Recuerdas quién era el personal de Kennedy?

White: No, realmente no en este momento. Brindé testimonio ante su comité del Congreso y probablemente hablé con alguien de su personal después. Se llevaron a cabo audiencias al respecto y logramos que se aprobara la legislación, como mencioné anteriormente. También hubo audiencias posteriores que el senador Bell de Maryland llevó a cabo. Uno de los problemas era dónde se ubicaría administrativamente el Centro Nacional para la Investigación de Servicios de Salud. ¿Sería parte del NIH?, ¿o debería integrar la sección que llamaban “del centro de la ciudad” de Salud, Educación y Bienestar?, ¿debería estar adjunto a la oficina del secretario?

Berkowitz: Entonces, ¿tendría que informar al secretario o al secretario adjunto?, ¿sería parte del Servicio de Salud Pública?, ¿esa era la idea?

White: Pensamos que cuanto más alto en la burocracia estuviera, mejores serían sus posibilidades de éxito. Eso nos lleva a un informe posterior. El comité asesor científico del presidente nombró un subcomité sobre Investigación y Desarrollo de Servicios de Salud que presidí. Después de un año de trabajo, emitimos nuestro informe en 1971. No llegó muy lejos porque hacia el final de nuestra asignación fuimos expulsados de manera sumaria del Antiguo Edificio de la Oficina Ejecutiva en un gran apuro. Eso fue cuando el Sr. Nixon estaba metiéndose en problemas, y nos trasladaron a otro edificio lateral y el informe, aunque fue impreso y distribuido de manera modesta, nunca recibió el tipo de discusión que esperábamos. Pero en ese informe argumentamos que el centro nacional debería estar ubicado de manera central y debería informar al secretario o al menos al secretario adjunto. Recuerdo que más tarde mantuve discusiones con Julie Richmond cuando era secretario adjunto sobre su ubicación y funciones.

Berkowitz: ¿Eso sería durante la administración de Carter? Él era secretario adjunto de Salud, Educación y Bienestar y Cirujano General. ¿Dónde estaba en la burocracia cuando Carter era presidente?, ¿estaba en algún lugar del Servicio de Salud Pública?

White: No, no creo que alguna vez haya estado en el Servicio de Salud Pública.

Berkowitz: ¿Entonces estaba simplemente flotando libremente en el Departamento?

White: Sí. Por supuesto, cayó en tiempos desafortunados debido a lo que algunos consideraron como políticas equivocadas de Paul Sanazaro; él fue el primer director del centro. Me había pedido que presidiera su comité asesor. Se llamaba la Junta Asesora Científica y Profesional para el Centro Nacional. Teníamos a algunas personas bastante destacadas, como Rosemary Stevens, David Mechanic y Bob Haggerty. Martin Feldstein también era miembro. Anteriormente intenté reclutarlo para mi departamento en Hopkins. Realizó una serie de excelentes estudios sobre atención médica cuando estaba en Oxford. Publicó en la revista *Medical Care* que mi amigo Bram Marcus había iniciado. Ahí es donde me enteré por primera vez del trabajo de Feldstein. Finalmente, John Dunlop lo contrató en Harvard. Herb Klarman estaba conmigo cuando lo entrevistamos en Hopkins. Teníamos un comité bastante bueno, pero no se tomó nuestro consejo. Recuerdo que Paul regresó de una visita con Wilbur Cohen, quien era secretario de Salud, Educación y Bienestar. Informó que Cohen le había dicho que había tres prioridades urgentes para el centro: reducir costos, mejorar la calidad y aumentar el acceso a la atención médica. Pregunté cuánto tiempo tenía el centro para lograr todo esto y Sanazaro dijo “*seis meses*”. Nuestro comité dijo que esto era imposible y que Cohen debería haber sido disuadido de la idea de que estos eran objetivos factibles a corto plazo. Desafortunadamente, Paul quería expandir rápidamente el centro. Uno de sus primeros movimientos fue hacer que la Oficina de Enfermedades Crónicas se transfiriera al centro; tenía una gran cantidad de subvenciones de segunda categoría que habían sido rechazadas por el NIH. Trataban sobre la gestión de enfermedades crónicas y sus servicios. Los estudios no estaban bien diseñados y había un tendal de burócratas que no eran muy agudos, pero la Oficina tenía mucho dinero en comparación con el presupuesto inicial asignado al Centro Nacional para la Investigación y Desarrollo de Servicios de Salud, como Sanazaro lo renombró. Aceptó a todas estas personas y trajeron consigo muchos más problemas con las subvenciones mediocres e incluso más con los burócratas descontentos, aunque sí obtuvo más dinero. Perdió el enfoque del centro en su totalidad y su misión, ya que se expandió demasiado rápidamente y de la manera equivocada. Bob Brook realmente podría decirte mucho sobre lo que sucedió porque era un miembro del centro durante este período. Ahora está en RAND Corporation y en la University of California. La otra persona altamente conocedora sería Tom McCarthy; fue director adjunto del Centro por un tiempo, y tiene un recuerdo total de casi todo. Conoce nombres, fechas, personas y actores. Ahora está semiretirado y vive en Potomac, Maryland. Valdría la pena hablar con él. Así que el centro cayó en tiempos difíciles durante la década de 1970. Prometió demasiado y no pudo cumplir. El Congreso se volvió cada vez más impaciente. Mi principio siempre ha sido prometer muy poco y entregar tanto como puedas antes de lo que tus patrocinadores esperan; sorprendes a las personas con algo que es útil y es útil rápidamente. Entonces el centro se metió en problemas conceptuales e incurrió en “dispersión”, es decir, asumió demasiados proyectos diferentes con poco enfoque y pocas prioridades. Todo esto llevó a problemas de credibilidad, y el presupuesto se redujo gradualmente a partir de mediados de la década de 1970. Se había evaporado bastante bien a principios de la década de 1980. Además, otro problema era que había una rivalidad poco saludable e innecesaria entre el Centro Nacional para la Investigación de Servicios de Salud y el Centro Nacional para las Estadísticas de Salud. Debes tener datos para hacer los análisis, y la pregunta es quién controla los datos. Luego hubo una división en tres partes con la Health Care Financing Administration (HCFA).

Berkowitz: Y antes de eso la Social Security Administration (SSA).

White: Ida Merriam había nombrado a Dorothy Rice en su equipo y Dorothy había contratado a Cliff Gaus. Recuerdo bien a Cliff Gaus en Hopkins, trabajando en su tesis una tarde alrededor de las 6:00 PM. Estaba realizando un estudio sobre la atención hospitalaria en el condado de Allegheny y Pittsburgh. Para ello, necesitaba datos de base poblacional sobre el alta hospitalaria para todo el condado, provenientes de Blue Cross y Blue Shield, Medicare y otras aseguradoras. Tenía un éxito moderado y grandes dificultades. Recuerdo que dijo algo como: “*Dios mío, si alguna vez tengo el control de esto, vamos a tener un sistema para documentar la atención hospitalaria, y me aseguraré de que se lleve a cabo*”. Bueno, sabía que este era su objetivo, y cuando Dorothy asumió el control del National Center for Health Statistics, le dije: “*Será mejor que traigas a Cliff Gaus aquí a tu equipo o se apropiará de todos los datos de alta hospitalaria y quizás otros datos de atención médica en la Health Care Financing Administration y ellos los controlarán*”. Ella dijo: “*Oh, no, Cliff es un buen tipo y yo lo contraté*”. Y yo dije: “*Claro que es un buen tipo, pero esto es lo que está haciendo*”. Así que había una lucha en curso entre el National Center for Health Statistics y su enfoque hacia las estadísticas vitales, es decir, contar a los muertos y demás, lo cual dije que está bien, pero hagamos algo por los vivos, y la Health Care Financing Administration controlando cada vez más los costos hospitalarios y los datos de alta hospitalaria, aunque el National Center for Health Statistics introdujo la *Encuesta Resumen de Alta Hospitalaria* y el *Conjunto Mínimo Uniforme de Datos* que se utilizó para recopilar los datos. Esa es otra historia interesante. Organicé una reunión internacional en 1967 en Airlie House para discutir una variedad de enfoques para recopilar información sobre las altas hospitalarias en un formato uniforme. Esta no era mi idea, Florence Nightingale la propuso en la década de 1860. Publicamos el informe de la conferencia en la revista *Medical Care* y en un volumen separado. Se incluyeron recomendaciones específicas para un *Conjunto Mínimo Uniforme de Datos* para la información de alta hospitalaria. Había unos 14 elementos que considerábamos importantes. Como mencioné anteriormente, el Sistema de Actividades Profesionales de Virgil Slee y uno o dos sistemas más pequeños habían recopilado, como solía decir el *New Yorker*, “*habitaciones llenas de datos sin que toquen el pensamiento humano*”. Recopilaron muchos datos, pero no produjeron mucha información utilizable. Así que promulgamos de forma independiente los resultados de nuestras deliberaciones y posteriormente persuadí a los funcionarios (creo que Ted Woolsey) en el National Center for Health Statistics para que establecieran un comité oficial para considerar nuestras iniciativas. Tardó 17 años para que el Gobierno Federal finalmente adoptara esta simple lista de 12 o 14 elementos sobre lo que les sucede a las personas cuando están en el hospital, quiénes son, cuál era su problema, qué se hizo por ellos, cuáles eran los costos, etc. La autoridad en este extraño ejercicio burocrático es Jim Cooley en la Universidad Estatal de Georgia, que ha seguido toda su historia de giros y vueltas y luchas jurisdiccionales y ha escrito varias piezas al respecto. Si no crees que la información es poder, necesitas averiguar por qué la American Medical Association y la American Hospital Association, varios hospitales y sus médicos, las compañías de seguros de salud y varias grandes burocracias federales y estatales estaban luchando para ganar el control de los datos. Y todavía continúa. Todavía no han resuelto los detalles veinte años después de nuestra conferencia en Airlie House y 130 años después de que Florence Nightingale propusiera la idea. Después de la conferencia sobre el resumen de alta hospitalaria, celebramos una análoga para datos de atención ambulatoria y luego una para datos de atención a largo plazo; ambos informes se publicaron también en *Medical Care* y en volúmenes separados. Volviendo al tema de la división en tres partes, no cabe duda de que el constante regateo entre la Health Care Financing Administration, el National Center for Health Statistics y el National Center for Health Services Research and Development no fue de absolutamente ninguna ayuda para este último. No tenía ningún control sobre las grandes sumas de dinero desembolsadas en Medicare y Medicaid y sus datos y no tenía control sobre el otro aparato estadístico y las encuestas nacionales subyacentes, todas controladas por el National Center for Health Statistics. Todo lo que el National Center for Health Services Research and Development podía hacer era llevar a cabo o encargar estudios *ad hoc* y encuestas especiales, y financiar proyectos iniciados por investigadores. Sugerimos en nuestro informe del comité asesor del presidente, *Mejorando la Atención Médica a través de la Investigación y el Desarrollo,* que las actividades estadísticas de estas tres burocracias se coordinaran, si no se consolidaran, de modo que al menos sus misiones individuales encajaran y no fueran operaciones completamente discretas no relacionadas. Sus datos e información deberían ser compatibles y admitir comparaciones y análisis. Para lograr eso, necesitábamos términos, definiciones, clasificaciones y estándares idénticos para el registro y la recopilación de datos. El National Center for Health Services Research and Development, sin control de ningún dato, entró en declive y literalmente desapareció del mapa burocrático y académico durante varios años hasta que fue resucitado como la Agency for Health Care Policy and Research.

Berkowitz: ¿Cuándo fue eso? ¿Quién era el presidente en ese momento?

White: No puedo estar completamente seguro; creo que Reagan. Cliff Gaus podría decirte.

Berkowitz: ¿Él fue el primer director?

White: No, había otro individuo, Clifton Jarrett, que fue el primer director. Parecía en gran medida ineficaz.

Berkowitz: Entonces Clifton Jarrett, Cliff Gaus y luego John Eisenberg.

White: Ese es mi recuerdo al respecto, sí. Jarrett fue el primer director.

Berkowitz: La Agency for Health Care Policy and Research es la sucesora del National Center. ¿Y está al nivel de agencia como la Health Care Financing Administration?

White: En la burocracia, creo que sí. Por cierto, Cliff también inició la Association for Health Services Research alrededor de 1981 o 1982.

Berkowitz: ¿De verdad? ¿También participaste en eso?

White: No, no lo hice. Me han hecho Miembro Honorario, pero no.

Berkowitz: ¿También esa revista comenzó por entonces?

White: Ayudé a iniciarla, pero eso fue algunos años antes de que se organizara la Association for Health Services Research. La American Hospital Association quería iniciar una revista llamada Hospital Research y yo estaba en la sección de Estudio de Servicios de Salud. La AHA solicitó una subvención para financiar el inicio de su nueva revista. También formé parte del equipo de visita in situ para negociar la subvención con la AHA. Dije: “*Es una gran idea comenzar esta revista, pero tiene que cubrir más que la investigación hospitalaria. Necesita llamarse algo como ‘Health Services Research’*”. Los funcionarios de la AHA discutieron de un lado a otro y pacientemente expliqué: “*Si quieren el dinero, tendrán que cambiar el enfoque*”. Así que se reunieron en la sala de atrás y regresaron diciendo que pensaban cambiar el enfoque. Así que les dimos la subvención y ayudamos a lanzar la nueva revista.

Berkowitz: ¿Cuándo fue eso?

White: Fue hace más de 32 años, así que eso lo situaría en 1964 o 1965.

Berkowitz: ¿Cuál es el grupo de interés para todas estas cosas diferentes? ¿Este Centro y esta Agencia? Tu departamento habría sido un grupo de interés, ¿Departamento de Organización de la Atención Médica o Política y Gestión de la Salud? ¿Había otros lugares que estaban haciendo esto? ¿Dirías que este es tu campo, la investigación en servicios de salud?

White: Supongo que sí. No me gusta ser categorizado. También estoy en el campo de la atención primaria/medicina familiar/medicina general. Estoy en el campo de la medicina interna. Estoy en el campo de las estadísticas de salud. Estoy en el campo de la epidemiología, especialmente en el campo de la epidemiología clínica. Estoy interesado en examinar problemas que afectan a todos los aspectos de la atención al paciente y en utilizar los mejores métodos disponibles para estudiarlos. Formalmente, supongo que soy un internista y epidemiólogo involucrado en la investigación de servicios de salud.

Berkowitz: ¿En dónde más, además de Hopkins, realizan esta investigación en servicios de salud?

White: La mayoría de las escuelas de medicina y escuelas de salud pública ahora tienen unidades que realizan este trabajo, aunque los títulos varían. Hay una membresía grande y en crecimiento en la Association for Health Services Research; ahora son un par de miles de individuos, creo. Su primera directora ejecutiva, Alice Hersh, falleció muy repentinamente el año pasado. Era una líder sumamente talentosa. Cliff Gaus lo inició, pero ella realmente lo puso en el mapa. ¿Estás familiarizado con sus publicaciones? Hay otra revista global que ayudé a iniciar: *International Journal of Epidemiology*. Estuve en el consejo de la International Epidemiological Association durante unos 15 años, más tiempo que cualquier otra persona, creo, como su tesorero y luego presidente. La asociación se inició alrededor de 1954 con una pequeña reunión en Nordwyck, Países Bajos; la Fundación Rockefeller financió las primeras reuniones y también muchas posteriores. John Pemberton, profesor de Medicina Social en Queens University, Belfast y Mickey Willard, un médico sumamente creativo que trabajaba en un hospital rural en Maine, fundaron lo que inicialmente llamaron el International Corresponding Club. Su propósito era promover el intercambio de ideas entre académicos jóvenes interesados en ampliar los horizontes de la medicina; la mayoría de ellos tenían becas de viaje del Commonwealth Fund o de la Fundación Rockefeller para visitar el Reino Unido y Europa o América del Norte. Pemberton y Willard publicaron inicialmente un boletín mimeografiado durante varios años. En 1971 negocié un contrato con Oxford University Press para comenzar la publicación formal del *International Journal of Epidemiology* y ha ido expandiéndose y prosperando desde entonces.

Berkowitz: Entonces, esto es algo incoherente pero aún un gran campo. Tiene economistas, tiene epidemiólogos...

White: ...tiene sociólogos, estadísticos y cualquiera que esté interesado. La red INCLEN que inicié cuando estaba con la Fundación Rockefeller ahora tiene 55 unidades de epidemiología clínica en 25 países.

Berkowitz: ¿Y eso qué significa?

White: Son las siglas para International Clinical Epidemiology Network (INCLEN). Es realmente una iniciativa de investigación en servicios de salud. Cada una de las unidades componentes de una escuela de medicina en el mundo en desarrollo consta de unos cuatro o cinco médicos capacitados en epidemiología, unos dos estadísticos de salud, un economista de la salud y un sociólogo, todos capacitados en conceptos y métodos epidemiológicos. Todos obtienen títulos de Máster en Ciencias en seis universidades del mundo desarrollado: McMaster University y la University of Toronto en Canadá; la University of Pennsylvania y la University of North Carolina en Chapel Hill en este país; y la University of Newcastle en Australia. John Eisenberg fue muy influyente para agregar economistas de la salud a cada unidad; algunos fueron capacitados inicialmente en la Wharton School en Penn. Así que tenemos este grupo de unos diez jóvenes profesores de escuelas de medicina en cada una de estas unidades, generalmente con sede en un departamento clínico y patrocinados por el decano de la escuela o el presidente de un departamento clínico. Están entrelazando preocupaciones y problemas de equidad, eficacia, efectividad y eficiencia en la atención médica en todo el mundo. Su trabajo, por lo tanto, es realmente otra forma de investigación en servicios de salud. Es la misma estructura básica diseñada para cambiar el pensamiento sobre políticas y prácticas de salud por parte de educadores médicos, políticos y el público. Estas unidades proporcionan el clima para hacer preguntas tan impertinentes pero importantes como: ¿Lo que estamos haciendo para los individuos o la comunidad hace más bien que mal? ¿Estamos respondiendo adecuadamente no solo a las necesidades individuales sino también a las necesidades de la población? ¿Qué esperan y requieren colectivamente los individuos y el público? Después de todo, son las personas las que sufren y pagan las facturas. Tienen derecho a tener una gran influencia sobre qué tipo de servicios se van a proporcionar. Todo es parte de un tema, no tanto tratar de establecer una disciplina, aunque algunas personas lo están haciendo. Quieren establecer juntas de certificación y reglas para entrar y reglas para mantener a otros fuera. Tuvimos grandes peleas en la International Epidemiological Association sobre quién es un epidemiólogo. Había quienes querían que todos pasaran por un elaborado proceso de selección para mantener una organización elitista. Les Breslow y yo pensamos que si la International Epidemiological Association podía acomodar a los tipos de enfermedades infecciosas y a los tipos de enfermedades crónicas, debería poder acomodar a los tipos de investigación en servicios de salud. Fuimos más allá y argumentamos que cualquier persona que estuviera lo suficientemente interesada como para pagar la cuota anual de membresía de U$S10.00 y pudiera escribir su nombre debería ser elegible para unirse. ¿Cuál es el punto de tener una organización elitista? No necesitamos ese tipo de cosa. Nuestras opiniones prevalecieron finalmente. Así que ahora tenemos dos o tres mil miembros de todo el mundo.

Berkowitz: Entiendo. Hablemos solo un minuto acerca de tu carrera. En 1976 dejaste Hopkins. ¿Cuál fue la razón de eso?

White: Para trabajar en el informe del comité asesor de ciencia del presidente, me tomé un año sabático de Hopkins. Había estado en el cargo dirigiendo este nuevo departamento durante aproximadamente siete años, así que decidí renunciar y volver a la vida civil.

Berkowitz: ¿Quién te reemplazó?

White: Phil Bonnett. Había sido profesor titular en el departamento. Luego recibí varias ofertas para decanatos, pero nunca aspiré a ser decano, especialmente de una escuela de salud pública. Tenía unas siete ofertas, una de una facultad de medicina y todas las demás de escuelas de salud pública. La University of California en Berkeley y la University of Pittsburgh fueron las que más me interesaron. Además, la Corporación RAND me ofreció el cargo de jefe de su programa de salud. En cualquier caso, pensé que tal vez debería hacer algo diferente. El United Hospital Fund en New York se me acercó y me ofreció un puesto como director de su nuevo Instituto para la Política de Atención Médica. Un tal Joe Terenzio dirigía el United Hospital Fund. Paul Densen me había recomendado para el trabajo. Densen era un estadístico de salud muy talentoso y uno de los pioneros en investigación de servicios de salud. Trabajó durante mucho tiempo con Sam Shapiro en el Health Insurance Plan of New York (HIP) y luego se fue a Harvard para establecer su Centro de Investigación de Servicios de Salud.

Berkowitz: Otro lugar pionero para la investigación de servicios de salud fue el Health Insurance Plan of New York

White: Oh, sí, y muy bueno. Sam es un excelente ejemplo de cómo te conviertes en un experto. Hasta donde puedo decir, hay tres formas: curso largo, curso corto y autoproclamación. ¡La mayoría de los expertos que conozco ahora trabajan en un campo para el cual no fueron entrenados! Son expertos por la tercera vía, la autoproclamación; aunque estoy seguro de que Sam nunca lo dijo. Sam solo tenía una licenciatura, pero es muy respetado como un investigador muy innovador y hábil. Lo recluté para Hopkins como director de nuestro Centro de Investigación de Servicios de Salud. De todos modos, Terenzio y su junta me ofrecieron este trabajo como director de su nuevo Instituto para la Política de Atención Médica. Lo acepté porque ofrecía un grado de independencia, la oportunidad de comenzar algo nuevo, un presupuesto sustancial y la capacidad de otorgar pequeñas subvenciones para proyectos valiosos. A mi esposa y a mí nos gustaba la idea de vivir en New York porque teníamos una hija allí. Sin embargo, no pasó mucho tiempo antes de que me desilusionara con todo el entorno.

Berkowitz: ¿Tenías que vivir en New York?

White: Sí, nos mudamos a Manhattan. Era amigo de Bob Ebert y él me recomendó para un trabajo en la Fundación Rockefeller, donde él era miembro de su junta directiva.

Berkowitz: ¿Te refieres a Bob Ebert del Milbank Fund y anteriormente decano de la Escuela de Medicina de Harvard?

White: Sí. En cuanto a Terenzio, supongo que la mejor frase para describir su comportamiento sería lo que Lady Astor solía describir como “inexactitud terminológica”. Nos separamos. No me gustaba su estilo. Por ejemplo, quería nombrar a un par de personas y a él no le gustaba la idea. Su junta parecía interesada solo en proteger las reputaciones de los hospitales de New York. Se oponían a hacer preguntas sobre cómo funcionaban. Uno de los estudios que estábamos planeando, pero que nunca se implementó, estaba diseñado para determinar cuántas radiografías del tracto gastrointestinal inferior eran “positivas” y detectaban enfermedades en comparación con las que eran “negativas” o normales. Creo que había leído en algún lugar que había una cuestión sobre si eran a) útiles y b) cuántas de ellas eran negativas. Así que íbamos a examinar esa cuestión con varios de los departamentos de radiología de los hospitales de New York. Creo que algunos de los radiólogos se asustaron y llegaron a ciertos directivos del United Fund, y ellos a su vez parecían generar una gran cantidad de interferencia política. Querían que hiciera cosas que impulsaran a los hospitales locales y los apoyaran. Yo estaba más inclinado a investigar y examinar cosas. Este no era el tipo de ambiente en el que podría prosperar y ellos, estoy seguro, no querían que hiciera ese tipo de investigación. El presupuesto también fluctuó hasta que consideré que era hora de irme. John Knowles, presidente de la Fundación Rockefeller, y yo habíamos tenido numerosas discusiones a lo largo de los años sobre la brecha entre la medicina y la salud pública y lo que podría hacerse para mejorar la comprensión y las relaciones. Las dos culturas se estaban alejando cada vez más. De hecho, Knowles me había entrevistado como posible director de Ciencias de la Salud, pero retiré mi nombre porque me pareció que querían a alguien con más experiencia en enfermedades tropicales de la que yo tenía. Finalmente, Ken Warren fue nombrado director. Luego, John Knowles me nombró como director adjunto y tuve aproximadamente la mitad del presupuesto de la División de Ciencias de la Salud para iniciar la Red Internacional de Epidemiología Clínica. Estaba mucho más en línea con lo que quería hacer.

Berkowitz: ¿Y realizaste este proyecto que estás describiendo? ¿Te quedaste allí hasta 1984 y luego decidiste retirarte esencialmente?

White: Sí. De hecho, Rockefeller tenía una jubilación obligatoria a los 65 en aquellos días, pero me mantuvieron hasta los 67.

Berkowitz: Es un tiempo considerable. Son 14 años. ¿Has estado haciendo consultoría?

White: Sí, he hecho bastantes cosas. La mayoría de las consultas implicaron semanas, meses o un año o dos en un caso, e incluyeron múltiples visitas y mucho viaje. Realicé una revisión para la División de Medicina General de la University of Iowa, otra revisión para la Academia Americana de Pediatría de su programa de investigación, y un estudio amplio del College of Medicine de la University of Saskatchewan y su relación con la población de la provincia y con los servicios de salud. También realicé consultoría estadística y de información de salud para UNICEF, el Programa de las Naciones Unidas para el Desarrollo y la Organización Panamericana de la Salud. He tenido dos períodos sustanciales en Australia. El primero fue para el Departamento de Salud de Australia del Sur. Involucró una evaluación de su programa de promoción de la salud, un asunto bastante lamentable. Una segunda revisión más extensa se realizó para el Ministro Federal de Salud, Neal Blewett. Visité todas las facultades de medicina del país, varios institutos de investigación y su única escuela de salud pública examinando el tipo de enseñanza. El efecto neto fue mi recomendación de que el gobierno cerrara la Escuela de Salud Pública, aumentara el presupuesto para la investigación en salud pública y redistribuyera el dinero a las facultades de medicina del país para mejorar la enseñanza de la salud pública. Todas mis sugerencias fueron aceptadas. Hubo una gran protesta y mucha infelicidad por parte de la fraternidad de salud pública, pero revisiones independientes posteriores de mi informe y las decisiones políticas tomadas han demostrado que la nueva forma de organización ha aumentado el interés en la salud pública y el número de personas involucradas en ella, y han mejorado la formación en salud pública. También sugerí establecer un Centro Nacional para Epidemiología y Salud de la Población en la Australian National University en Canberra. También ha tenido un rendimiento excepcional. De hecho, acabo de recibir una notificación de que están celebrando su décimo aniversario este otoño. Bob Douglas, el director, ha hecho un excelente trabajo. He ido a China como consultor con un equipo del Banco Mundial revisando su solicitud de fondos para desarrollar aspectos de sus servicios de salud. Más tarde regresé a petición del gobierno chino para analizar, junto al Ministerio de Salud chino, el establecimiento de algo similar al US National Center for Health Statistics. Ambas visitas implicaron una cantidad considerable de viajes a China. Dos otras visitas allí también me permitieron ver gran parte de ese inmenso país y observar los cambios extraordinarios que están teniendo lugar. He escrito *Healing the Schism: Epidemiology, Medicine, and Public’s Health*, a petición de la Fundación Rockefeller. Edité otro volumen: *The Medical School’s Mission and the Population’s Health* y escribí un par de capítulos para él. Ese libro se basó en una conferencia internacional que organicé para la Royal Society of Medicine y la Fundación Josiah Macy Jr. También he escrito unos 50 artículos desde que me retiré y todavía formo parte de tres o cuatro comités editoriales. En la Emory University hay un Instituto para la Investigación de Servicios de Salud que lleva mi nombre y soy uno de los directores eméritos. David Ballard es su presidente. Su formación en economía, medicina interna y epidemiología es similar a la mía, por lo que tenemos mucho en común. El Instituto está teniendo un rendimiento excepcional.

Berkowitz: ¿Por qué vinieron a Charlottesville?

White: Teníamos un lugar aquí en el campo que empezamos a construir en 1978. Lo usábamos para vacaciones, o si yo estaba fuera en un viaje largo, Isabel venía aquí. Luego nos mudamos aquí a tiempo completo en 1984.

Berkowitz: ¿Simplemente les atraía esta área?

White: Tengo un hermano que es parte del profesorado aquí, así que esa fue una de las principales atracciones.

Berkowitz: ¿Cuál es su campo de estudio?

White: Durante muchos años fue presidente del Departamento de Psiquiatría de la University of Virginia, pero su principal preocupación es el estudio de la reencarnación. De hecho, creo que acaba de regresar de un viaje de campo a la India anoche. Recientemente publicó una monografía en dos volúmenes en la que documenta cientos de casos de niños que recuerdan vidas pasadas. Tiene informes de casos en los que hay fotografías e informes médicos de marcas de nacimiento y de las heridas correspondientes que los niños afirman haber sufrido en una vida anterior. Ha podido documentar de manera independiente registros médicos sin que los individuos lo sepan, sobre lo que les sucedió a estas personas en sus supuestas vidas anteriores. Hay una versión más corta de su trabajo, realmente un resumen de todos sus hallazgos que también se publicó recientemente. Acaba de encontrar otro caso en Chicago hace uno o dos meses; es bastante notable. Creo que espera intentar publicarlo en *JAMA*, pero las revistas establecidas no están muy interesadas en su línea de investigación. No encaja en el paradigma predominante. La mayoría de sus casos están en India, Birmania, Sri Lanka, Turquía y entre los indios del noroeste, Columbia Británica, Washington y Alaska. Ese es el tipo de trabajo que realiza.

Berkowitz: Eso es interesante. Permítame hacerle una última pregunta. Ha hecho todo este trabajo heterogéneo y no le gusta ser encasillado y realmente no cree en la especialización. ¿Cuál diría que es su obra más representativa? ¿Sería ese artículo sobre ecología? ¿Cuál sería la respuesta si alguien preguntara: “¿Cuál es el legado de Kerr White al campo de la investigación en servicios de salud?” ¿Cuál sería la respuesta?

White: Creo que ayudar a los clínicos y médicos en general a pensar de manera más creativa sobre su misión y sus actividades y adoptar un paradigma más amplio que incluya las necesidades de toda la población y abarque no solo la perspectiva biomédica, sino también la clínica y poblacional. Ese paradigma reconocería que al menos la mitad del ámbito de la atención médica está relacionada tanto con el componente de cuidado como con el de curación. Este tipo de pensamiento se vuelve complejo y requiere un cambio de actitud desde las nociones reduccionistas, moleculares y unicausales de la enfermedad que parecen dominar el paradigma predominante, para usar una palabra trillada, hacia una visión más amplia de toda la cuestión. Está bien, puedes hacer tu investigación reduccionista y puedes hacer tus trasplantes de córnea e hígado, pero al menos comprende que aquí hay mucho más en juego y hay muchas otras perspectivas y muchas otras necesidades. Lo que necesitamos urgentemente son sistemas de atención médica equilibrados y carteras equilibradas de investigación para avanzar realmente con los problemas de toda la población. Prefiero estas aspiraciones a las nociones de que mapear el genoma y darle a todos una tarjeta inteligente con su derecho de autor genético, o limpiar el medio ambiente, son suficientes para brindar buena salud y mejorar la vida de todos. Eso es complicado, realmente no puedo resumirlo en una palabra. La gente se pregunta cómo puedes estar involucrado en esto o en aquello, pero yo los veo a todos como partes de un todo. Veo mis pequeños esfuerzos como un estímulo para una faceta del conjunto y luego fomento otra faceta. Creo que necesitamos información mejorada sobre salud, enfermedad y servicios de salud. Y eso incluye información sobre el ubicuo “efecto placebo”, la intervención terapéutica más poderosa que poseen los médicos, así como información sobre píldoras, pociones y procedimientos. Hemos oído hablar de la sabiduría que hemos perdido en el conocimiento, y del conocimiento que hemos perdido en la información, pero lo peor de todo es lo que se ha perdido en los datos. Los burócratas y otros siguen hablando de recopilar más datos. Lo que necesitamos es mejor información obtenida de los datos, y luego necesitamos transformar la información en inteligencia y, sobre todo, en sabiduría para guiarnos. Eso parece escasear en la labor médica. Reconozco que todo eso es bastante complicado, pero no estoy seguro de que estas cosas puedan reducirse a un fragmento de sonido.

Berkowitz: Me parece que en su caso tiene un legado intelectual que está involucrado en cambiar la gestalt de alguna manera de la práctica de la medicina y del encuentro clínico. También tiene su legado pedagógico, estas personas que han sido sembradas por usted en el campo. Y está el legado organizativo también. El hecho de que la burocracia federal ahora comprenda, de alguna manera vaga, este concepto. Eso es un papel bastante importante.

White: Intenté unirlos. La mayoría de estas ideas no son tan nuevas. En *Healing the Schism: Epidemiology, Medicine, and the Public’s Health* intenté cubrir algo del contexto histórico. Crecimos políticamente informados. Mi padre era periodista, el corresponsal canadiense para el *Times* de Londres, el *Economist* y varios periódicos estadounidenses. De hecho, solíamos recibir el *Baltimore Sun* todos los días.

Berkowitz: En aquel entonces era un periódico realmente excelente.

White: Sí, lo era. Era un periódico estupendo. Y el *Boston Transcript*. Así que crecimos con muchos visitantes en un entorno político altamente crítico. La mitad de ellos eran británicos y la otra mitad estadounidenses, así que uno se acostumbraba a traducir ideas sociales, políticas y económicas de un lado a otro. El mejor amigo de mi padre había establecido lo que entonces era la Dominion Bureau of Statistics y ahora se llama Statistics Canada, así que en casa solíamos recibir una avalancha de informes estadísticos nacionales e internacionales. No pretendí entenderlos cuando era joven, pero las estadísticas nos rodeaban todo el tiempo, por lo que mi interés en ellas se estimuló. Incidentalmente, trabajé con Martin Wilk, un exdirector de Statistics Canada. Preparamos un artículo bastante extenso sobre sistemas de información de salud que describe lo que debería producir un sistema de información de salud verdaderamente integral. Nunca se publicó. Lo desarrollamos para el Canadian Institute for Advanced Research en Toronto cuando ambos éramos miembros de su Comité Asesor para su Programa de Salud de la Población.

Berkowitz: ¿Hay algo más que le gustaría mencionar, algún tema que no hayamos cubierto? Esto estuvo muy bien. Ayuda a dar su esencia.

White: Otro hito interesante fue la reunión de 1970 del Comité de Expertos en Estadísticas de Salud de la OMS. John Brotherston, a quien mencioné anteriormente, era el decano de la Facultad de Medicina de la Universidad de Edimburgo y más tarde el director médico de Escocia y profesor de Medicina Social. Creo que probablemente fue el mejor funcionario de salud que encontré en cualquier lugar de cualquier país. Presidió el Comité de Expertos de la OMS y yo fui su relator. En esa reunión definimos, hasta donde yo sé por primera vez, los términos “eficacia”, “efectividad” y “eficiencia” para evaluar programas de salud. Las importantes distinciones entre los tres términos provinieron en gran medida de dos personas. Uno era Sakari Haro de Finlandia, quien probablemente sabe más sobre información en salud que cualquier otra persona que haya conocido. El otro era un economista de la salud francés, George Roches, quien escribió un excelente libro en francés sobre economía de la salud y lamentablemente falleció hace algunos años. Su descendiente intelectual, Simone Sandier, dirige CREDOC, que es una importante operación internacional de estadísticas de salud en París. Ella es una gran amiga de Uwe Reinhardt y es miembro del Institute of Medicine. Haro y Roches juntos propusieron estas distinciones, particularmente entre eficacia y efectividad, que con demasiada frecuencia se confunden. La eficacia se refiere a la evaluación de una intervención médica basada en un estudio controlado, aleatorizado y doble ciego. La efectividad se refiere a la evaluación del impacto de una intervención supuestamente eficaz en el mundo real con médicos reales que pueden o no recetar la intervención adecuadamente para pacientes reales con diversas manifestaciones de su enfermedad y que pueden o no tomar la intervención prescrita adecuadamente. La eficiencia se refiere al uso prudente de recursos para obtener de manera óptima los resultados deseados. Hasta donde yo sé, esa fue la primera vez que se definieron estos términos importantes. Creo que ahora estamos aclarando la literatura entre eficacia y efectividad y los veo utilizados con mucha más precisión, especialmente por la Cochrane Collaboration. Nuevamente, estas ideas surgieron cuando un francés y un finlandés las debatieron y el resto de nosotros las interiorizamos y yo las redacté en el informe, pero no generé las ideas. Las ideas provienen de muchas fuentes diferentes y no son propiedad de ninguna disciplina o individuo en particular. Uno era un médico convertido en estadístico de salud y el otro era un economista de la salud. Desde 1964 hasta 1976, nuestro departamento en Hopkins lideró un gran estudio internacional patrocinado por la OMS sobre el uso de la atención médica. Se llamaba *International collaborative study of medical care utilization* (ICS-MCU). Lo que nos propusimos hacer fue observar la distribución del uso de servicios de salud en poblaciones definidas. Estudiamos siete áreas en doce países: Argentina, Gran Bretaña, Canadá, Finlandia, Polonia, Yugoslavia y Estados Unidos. Nuestros equipos, todos entrenados con los mismos estándares, realizaron entrevistas domiciliarias con muestras de probabilidad de aproximadamente 1.000 hogares en cada área de estudio y casi 48.000 personas en total; la tasa de respuesta general fue del 96%, bastante inusual para una investigación de este tipo, pero lo planificamos con mucho cuidado. Encontramos que los patrones de uso eran bastante similares en las diferentes áreas de estudio y países. Barbara Starfield ayudó a escribir uno de los artículos más importantes que documentan claramente que cuando había atención primaria adecuada, es decir, médicos generales fácilmente disponibles, la utilización del hospital era mucho menor que cuando lo contrario era cierto, sin importar el país. La ley de Roemer establece que “una cama hecha es una cama llena”. Mostramos, creo que de manera concluyente, que no importa la cantidad de camas por cada mil habitantes; si había atención primaria adecuada y las personas tenían acceso a un médico general, el uso de camas de hospital era mucho menor. Todas estas eran tasas de población ajustadas y estandarizadas. Este enorme proyecto fue una gran experiencia de aprendizaje para todos los participantes; duró doce años.

Berkowitz: ¿Eso fue algo de Hopkins?

White: Comenzó en Vermont. George Silver me involucró en ello, pero yo era el presidente de la empresa. En 1976, Oxford University Press publicó un gran volumen de nuestros resultados titulado *Health Care: An International Study*. También publicamos alrededor de cien artículos. Varias tesis se basaron en nuestros hallazgos y se realizaron análisis de los datos originales no solo en Hopkins, sino también en otras universidades aquí y en el extranjero. Hicimos disponibles cintas de los registros para aproximadamente una docena de universidades. Tuvimos consultores maravillosos, Ozzie Sagen, el brillante director asociado del National Center for Health Statistics y mi mejor amigo, y Charlie Cannel de la University of Michigan’s Social Survey Center. Nos enseñamos mutuamente. Aprendí mucho de los finlandeses, polacos y británicos, especialmente.

Berkowitz: Esa es otra faceta tuya, ¿verdad?

White: Sí, siempre he sido un internacionalista. Otra gran tarea fue el volumen que hice, titulado *The task of medicine*, publicado en 1988. Charles Odegaard, expresidente de la University of Washington, escribió un libro seminal titulado *Dear Doctor* en el que básicamente reprendía a la profesión médica por su fracaso en escuchar a sus pacientes y realmente “cuidar” de ellos. Fue patrocinado por la Kaiser Family Foundation y se distribuyó ampliamente. Escribió sobre la necesidad de una mejor comunicación, una mejor comprensión entre pacientes y médicos; algunas de las cosas de las que hemos estado hablando. Cuando Al Tarlov, presidente de la Kaiser Family Foundation, celebró una reunión en Wickenburg, Arizona, para discutir el libro de Odegaard, me pidió que fuera el relator. Escribí un artículo largo revisando y relatando los temas discutidos y, junto con varios anexos de los documentos más interesantes presentados en la reunión, se publicó como *The task of medicine: Dialogue at Wickenburg*. El volumen fue bien recibido y hubo miles de solicitudes para él. El jefe del Departamento de Salud de New York, que murió recientemente, David Axelrod, ordenó copias para todos los estudiantes de medicina de primer año en el estado de New York. Kaiser distribuyó al menos diez mil copias. Eso no es un *bestseller*, pero para libros médicos académicos es más que satisfactorio. Por supuesto, se distribuyeron de forma gratuita, por lo que eso marca una diferencia sustancial. Según su productor, con quien me reuní varias veces, *The task of medicine* fue la inspiración para un documental de ocho partes filmado por un consorcio internacional de cadenas de televisión nacionales, incluidos la BBC, PBS, la televisión australiana y española, entre otros. La serie se llamaba *Medicine at the crossroads*, pero tenía un título diferente en Gran Bretaña. Tuve un pequeño papel en las secciones que trataban sobre Hopkins. También fui editor en jefe de un volumen para la Organización Panamericana de la Salud: *Health services research: An anthology*, para el cual seleccionamos cien artículos. Habíamos recopilado nominaciones de un gran número de personas de todo el mundo. Barbara Starfield estaba en el comité editorial que presidía, y redujimos los candidatos originales a cien artículos después de varias iteraciones de la lista entre un grupo de expertos. El volumen parece haber sido bien recibido. Otra empresa en la que estuve involucrado fue la Ambulatory Sentinel Practice Network (ASPN). Lo patrociné mientras estaba en Rockefeller y ahora está funcionando muy bien. La red consta de más de 100 profesionales, principalmente médicos de familia, pero también algunos internistas y pediatras, principalmente de Estados Unidos y algunos de Canadá. Seleccionan un problema médico discreto, como dolor de cabeza, dolor de oído en niños, aborto espontáneo, síndrome del túnel carpiano o depresión, por ejemplo, y cada clínico participante registra en un formulario sencillo datos esenciales sobre el encuentro. Colectivamente, la red puede examinar la distribución del problema y las intervenciones diagnósticas y de tratamiento realizadas. Nuevamente, esta es otra forma de averiguar qué sucede en los consultorios médicos. Hasta la fecha, este método ha tenido bastante éxito. Se modeló a partir de redes similares en Gran Bretaña y Holanda. Muchos pensarían que estos eran desarrollos estadounidenses, pero pensé que estas eran buenas ideas originadas en el extranjero y patrocinamos un par de las reuniones iniciales de ASPN cuando estaba en Rockefeller. Hay muchos enfoques diferentes para desarrollar un campo. Otro volumen que hicimos cuando era presidente de la International Epidemiological Association fue *Epidemiology as a fundamental science: Uses in planning, administration, and evaluation*. Oxford University Press también lo publicó. Debe haber salido alrededor de 1976. Se vendió muy bien y se agotó hace aproximadamente un año. En un capítulo hice algunos cálculos, estimando cuántos epidemiólogos se necesitarían en Estados Unidos. Hace un par de años vi una referencia a mi estimación y ahora se consideró una subestimación sustancial, aunque en aquel momento algunos consideraron que mis números eran una sobreestimación brutal. Aparentemente, nuestro volumen se usó como libro de texto en varias escuelas de medicina. No tomamos regalías para que el libro fuera más económico. Hay muchas formas diferentes en las que se puede abordar la difusión de ideas. La mayoría de ellas parecen funcionar la mayor parte del tiempo. Una de las cosas que me preocupa mucho en este país, y esto es cierto no solo en los campos que me interesan, es la falta de memoria institucional. Es especialmente generalizado en la burocracia. La gente llega y piensa que la historia comenzó cuando asumieron el cargo. Parece haber poca conciencia de lo que otras personas han contribuido y de lo que ha ocurrido antes. Como dice un amigo mío, “*si ves una tortuga en un poste de la cerca ¡sabes que no llegó allí sola!*”. Hay muy pocas ideas nuevas y buenas, especialmente en la atención médica. La mayoría de las ideas nuevas no son tan buenas y la mayoría de las ideas buenas no son tan nuevas. En la escuela de medicina de McGill teníamos un maravilloso profesor de historia de la medicina, Andrew McPhail, y también teníamos un muy buen profesor de fisiología, Hebel Hoff, quien luego se trasladó a la Baylor University en Texas. Solía dramatizar muchos de los principios esenciales de la fisiología con demostraciones de los experimentos originales que ilustraban el descubrimiento fundamental. Ambos maestros ayudaron mucho a fomentar mi respeto por la historia médica y los logros pasados de los pioneros. Supongo que crecer en un hogar que tenía un enorme respeto por la historia y por la amplitud y el alcance de las experiencias humanas también marcó la diferencia. Todas estas exposiciones dejaron una impresión duradera en mí. Por ellos estoy eternamente agradecido. Tendremos que ver qué viene en el futuro.

Berkowitz: Sí, muy bien. Esa es una buena nota con la que terminar.

White: Gracias.

